# Mass development of a filamentous and likely nitrophilous aerophytic green alga on tree bark: *Apatococcus ammoniophilus* sp. nov. (Chlorophyta, Trebouxiophyceae)

**DOI:** 10.3389/fmicb.2025.1633308

**Published:** 2025-07-23

**Authors:** Ulrik Søchting, Thomas Friedl, Øjvind Moestrup, Felix Grewe, Yukun Sun, Yağmur Tarhana Çakır, Markus Ganzera, Karin Glaser, Svenja Heesch, Fabian Hammerle, Daniel Nimptsch, Birgit Olberg, Ulf Karsten

**Affiliations:** ^1^Section Ecology and Evolution, Department of Biology, University of Copenhagen, Copenhagen, Denmark; ^2^Department Experimental Phycology and Culture Collection of Algae (EPSAG), Albrecht-von-Haller-Institute for Plant Sciences, Georg August University, Göttingen, Germany; ^3^Section Marine Biology, Department of Biology, University of Copenhagen, Copenhagen, Denmark; ^4^Grainger Bioinformatics Center, Field Museum, Chicago, IL, United States; ^5^Institute of Pharmacy/Pharmacognosy, University of Innsbruck, Center for Chemistry and Biomedicine, Innsbruck, Austria; ^6^Faculty for Chemistry, Physics and Biosciences, Biology/Ecology, Freiberg, Germany; ^7^Department of Applied Ecology and Phycology, Institute of Biological Sciences, University of Rostock, Rostock, Germany; ^8^Department of Maritime Systems, Interdisciplinary Faculty, University of Rostock, Rostock, Germany

**Keywords:** terrestrial epiphytic, amplicon-based metabarcoding, bioindication, metagenomics, *Hormidium crenulatum*, *Klebsormidium*, phylogenetic analyses, mycosporine-like amino acids (MAA)

## Abstract

**Introduction:**

A filamentous green alga forming significant biomass on twigs and needles was observed to have increased invasively in Denmark in recent decades. It was particularly abundant in coniferous plantations in western parts of Denmark that experience the highest modelled concentration of atmospheric nitrogen deposition. However, its species identity and taxonomy remained unknown.

**Material and methods:**

Selected algal samples from various substrates were analyzed for their ribosomal DNA sequences, metagenomic, and biochemical compounds [polyols and mycosporine-like amino acids (MAAs)].

**Results:**

Phylogenetic analyses revealed the alga’s position within the Trebouxiophyceae (Chlorophyta), forming an independent lineage within *Apatococcus*. Though it was associated with various other Trebouxiophyceae species, the metagenome showed exceptionally high coverage of the *Apatococcus* contigs, proving its predominance, consistent with the amplicon-based approach. The low molecular weight carbohydrates, arabitol, erythritol, and trehalose – with erythritol displaying the highest concentrations—were recovered. The presence of erythritol provided chemotaxonomic support for the classification in *Apatococcus*. Additionally, a unique UV-absorbing mycosporine amino acid (MAA), likely new for the Trebouxiophyceae, was found. The species is described here as *A. ammoniophilus*, and the observed morphological features leave no doubt that it has been recorded from Denmark more than a 100 years ago. Morphological features are shared with its closer relatives, such as the presence of a ring of particles surrounding the nucleus and the formation of two-celled units.

**Discussion:**

The presence of low molecular weight carbohydrates and the unique MAA in *A. ammoniophilus* well explain the biochemical basis for its aeroterrestrial lifestyle, as these organic compounds protect against desiccation and UV-radiation, respectively. Even though the genotype of *A. ammoniophilus* has also been found in inconspicuous biofilms devoid of filamentous stages on various substrates with presumably low ammonia deposition, the very invasive colonization in recent decades in western Denmark is assumed to be due to ammonia deposition. Consequently, *A. ammoniophilus* is suggested to be a potential biological indicator of air borne nitrogen deposition. A possible connection between filamentous growth and nitrogen accumulation needs further investigation, including culture experiments.

## Introduction

Eukaryotic microalgae and cyanobacteria inhabit numerous terrestrial habitats across all biogeographic regions, forming phototrophic biofilms at the interface between the atmosphere and natural or anthropogenic solid substrates. Consequently, these ecologically unique taxa are classified as aerophytic. In temperate regions of the Northern Hemisphere, eukaryotic green microalgae from the Chlorophyta and Streptophyta are the most abundant terrestrial microorganisms (Häubner et al., 2006; Rindi, 2007; Holzinger and Karsten, 2013). These aerophytic, phototrophic biofilms cover natural surfaces such as rocks, tree bark, soil, and plant surfaces, as well as anthropogenic substrates like concrete, glass, and metal (Karsten et al., 2007), often forming conspicuous communities.

Water availability is a key ecological driver for these aerophytic algae, which are poikilohydric organisms and thus cannot actively regulate their water content (Kranner et al., 2008). As a result, they can easily undergo desiccation stress under water-limited conditions, although they often display adaptive traits to survive partial or even complete water loss (Holzinger and Karsten, 2013).

Desiccation typically results in a significant decline in photosynthesis and growth, with both physiological processes potentially being completely blocked under severe stress conditions (Holzinger and Karsten, 2013). Bertsch (1966) investigated the impact of desiccation on carbon assimilation by reducing air humidity in the aeroterrestrial green alga *Apatococcus lobatus*. This taxon is among the most abundant green algae in temperate Europe, forming conspicuous biofilms on tree bark (Barkmann, 1958), roof tiles, and building surfaces. Bertsch (1966) demonstrated that these cell packets or biofilms maintain a hydration equilibrium with the vapor pressure of the air, showing optimum carbon assimilation at 97–98% relative air humidity (rah); carbon assimilation decreased to 50% at 90% relative humidity and ceased at 68% relative humidity.

Aerophytic microalgae have developed a variety of morphological, physiological, and biochemical adaptive traits to the harsh terrestrial environment (Holzinger and Karsten, 2013). Photosynthesis is a key physiological process and hence efficient control of light absorption and energy distribution in the photosynthetic apparatus during dehydration is crucial to minimize or prevent photoinhibition. The biochemical capability to synthesize and accumulate organic osmolytes is regarded as a fundamental biochemical mechanism to provide desiccation tolerance (Holzinger and Karsten, 2013). By accumulating such compounds, aerophytic microalgae maintain turgor, membrane integrity, and macromolecule structure by compensating for changes in water potential without incurring metabolic damage (Yancey, 2005; and references therein). Many aerophytic microalgae, such as *Apatococcus*, *Diplosphaera*, *Stichococcus,* and other members of the green algal class Trebouxiophyceae (Chlorophyta), synthesize and accumulate polyols like glycerol, arabitol, ribitol, mannitol, and sorbitol (Gustavs et al., 2011, 2016; Hotter et al., 2018; Medwed et al., 2021). Polyols can also act as antioxidants or heat protectants, leading to the stabilization of proteins (Karsten et al., 2007); due to their multiple functions, polyols serve as one of the biochemical explanations for compensating terrestrial stress conditions.

Besides desiccation, aerophytic microalgae are regularly confronted with solar ultraviolet radiation (UVR), which exerts many harmful effects on cells and can even cause DNA damage (Karsten, 2008). Hence, many aerophytic microalgae are capable of synthesizing and accumulating UV-absorbing mycosporine-like amino acids (MAAs), which are low-molecular weight compounds with numerous chemical variations in their side groups and substituents, resulting in different absorption maxima between 310 and 360 nm (Karsten, 2008; Hartmann et al., 2016, 2020; Hotter et al., 2018). These colorless sunscreens shield the algal cells from UVR by absorbing the harmful radiation energy and converting it into harmless heat (Bandaranayake, 1998).

In temperate regions, the bark of tree trunks is most often covered by lichens or green algal biofilms which are dominated by members of Trebouxiophyceae genera, e.g., *Apatococcus*, *Desmococcus*, *Diplosphaera*, and *Trebouxia* (Medwed et al., 2021; Karsten et al., 2022), as well as *Trentepohlia* (Ulvophyceae, Chlorophyta) (Holzinger et al., 2023).

**Figure 1:**
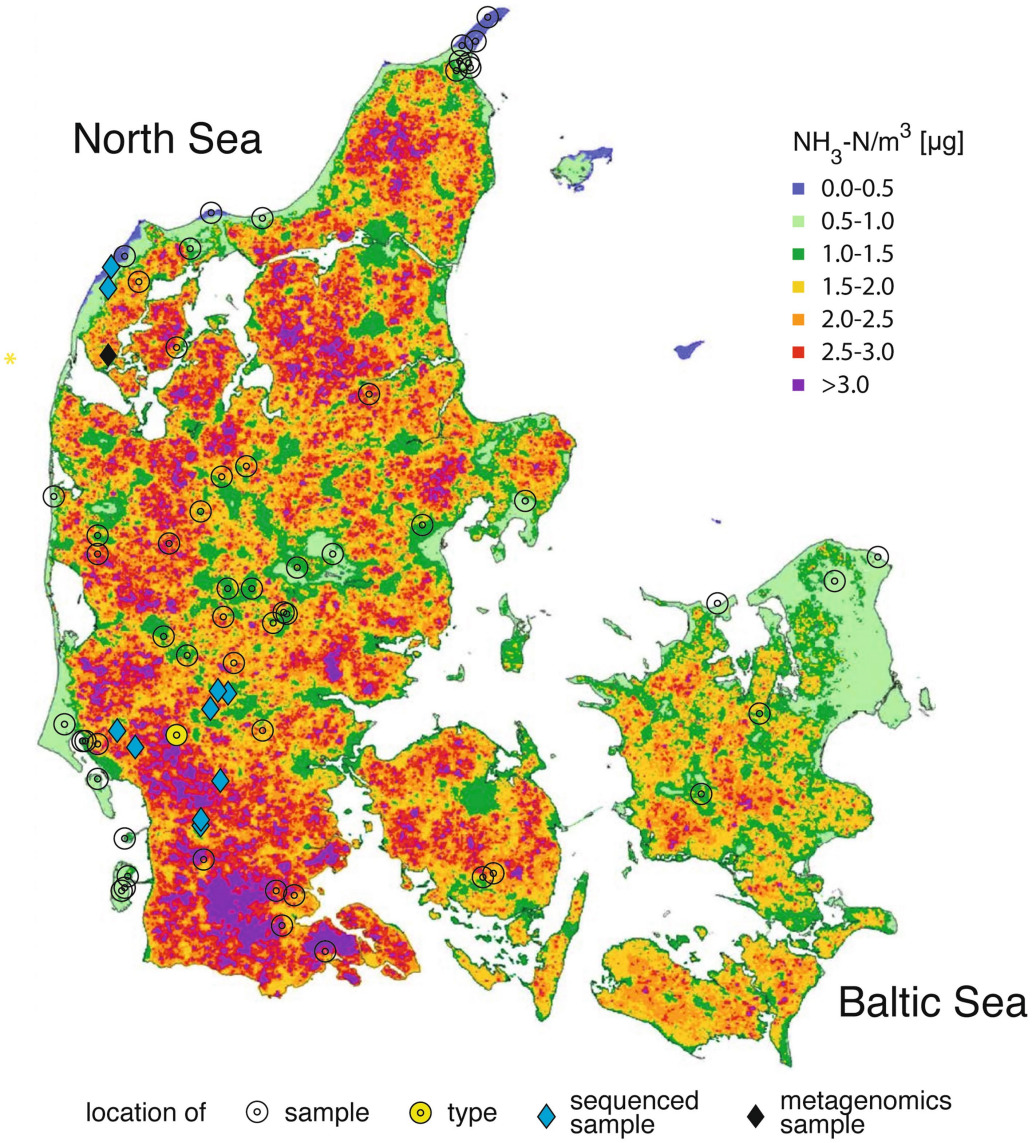
Locations from which the samples of the filamentous *Apatococcus ammoniophilus* have been collected (2018–2024; Table 1) superimposed on the map of Denmark with calculated annual mean values of ammonia-N concentration in the air in 2020 of Ellermann et al. (2021).

**Figure 2:**
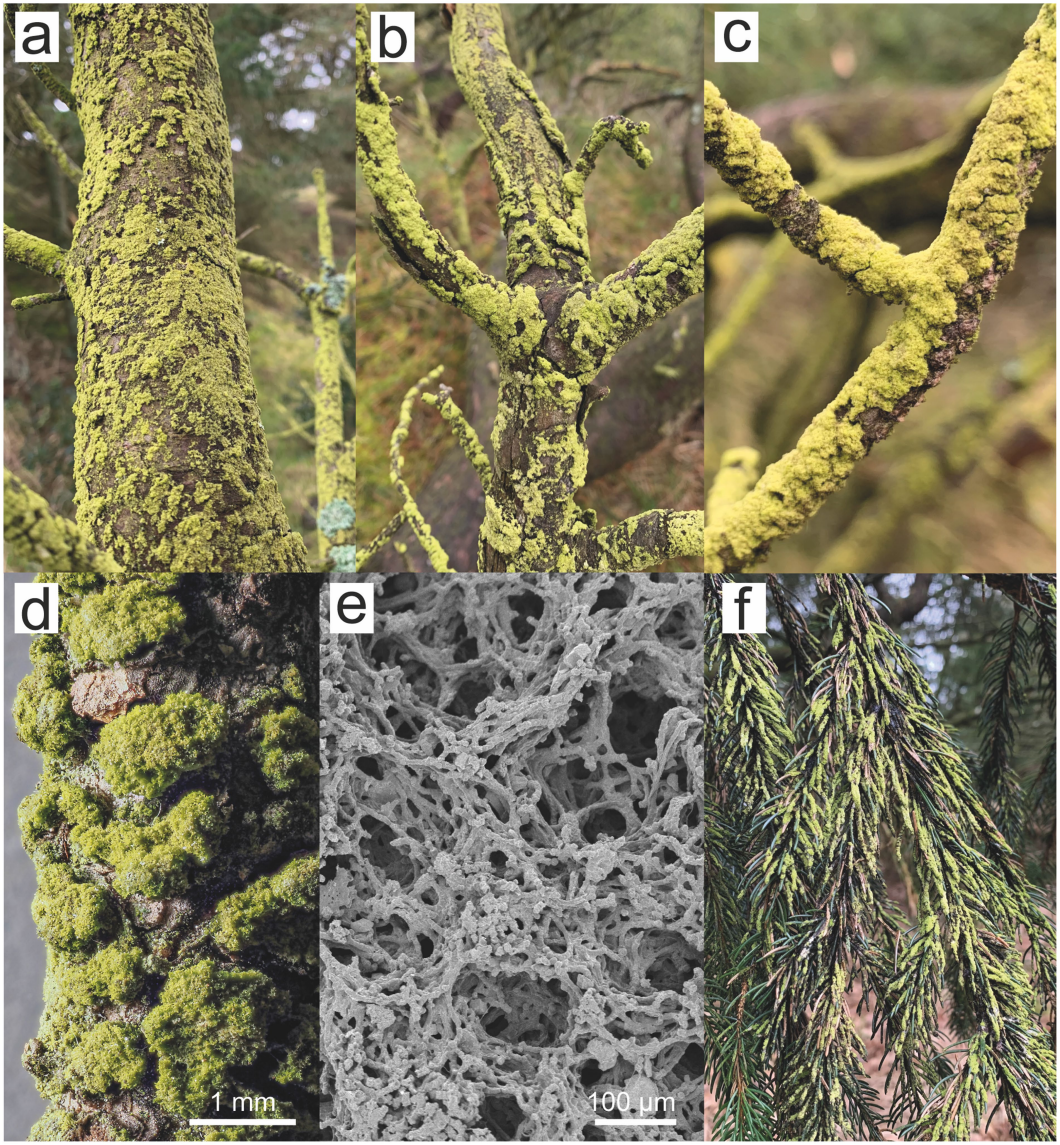
*Apatococcus ammoniophilus* biofilms on various tree substrates in Denmark. **(a,b)** On the trunk of *Pinus mugo*, Kikkebjerg, Fanø (voucher US13007). **(c,d)** On the twig of *Pinus* sp., Råbjerg Hede, Vendsyssel, Jutland (voucher US13044). **(e)** On the bark of *Pinus* sp., Mosevrå Kirke, Jutland, SEM (voucher US12952). **(f)** On the twigs and needles of *Pinus* sp., Løbners Plantage, Jutland.

In Denmark, the formation of pulverulent algal biofilms on the bark of trees is extremely common. However, in the 1980s, a filamentous alga suddenly became much more frequent, particularly in Jutland (Søchting, 1997) (Figure 1). This alga occupied the trunks, branches, dead twigs, and needles of conifers including Christmas trees, causing aesthetically unacceptable discoloration, which led to economic problems in selling these products. The occurrence of a filamentous green alga on the twigs of spruce in Denmark was already reported by Petersen (1915) in his studies on Danish ‘aerophilic’ algae. He identified it as *Hormidium crenulatum* and provided a detailed description and illustration of the species. He also observed that *H. crenulatum* was growing only where ammonia influence from farming was present, and its substantial expansion during the 1980s was suggested to result from increased ammonia deposition from husbandry farming (Søchting, 1997). In recent years, this alga has been recorded from all parts of Denmark, and in agricultural regions of Jutland it forms almost monospecific filamentous mats on coniferous and deciduous bark, as well as on spruce and fir needles and on dwarf shrubs in heathlands (Figure 2). *H. crenulatum* was transferred to *Klebsormidium* and lectotypified by Lokhorst and Star (1985) and later epitypified by Mikhailyuk et al. (2015). However, the morphology of the epitype of *K. crenulatum* differs from the Petersen (1915) alga (see Discussion below), raising serious doubts about whether the latter could be a member of Klebsormidiophyceae (Streptophyta). It follows that this abundant aerophytic filamentous green alga represents a species for which no name is yet available.

We studied a selection of environmental samples of the filamentous alga obtained from various substrates to analyze their ribosomal DNA sequences and perform a metagenomic community profiling, aiming to uncover the alga’s phylogenetic position. Additionally, we examined the samples for specific biochemical compounds, i.e., low molecular weight carbohydrates (polyols) and mycosporine amino acids (MAAs), which may form important adaptive traits for the alga’s subaerial lifestyle.

## Materials and methods

### Sampling

This study is based primarily on intensive collecting of bark, twigs, branches and needles of mostly pine trees in Denmark, with addition of material from Germany, Norway, Sweden, England and the Netherlands. Studied specimens are listed in Table 1. Vouchers of all collections are deposited in the Natural History Museum of University of Copenhagen (C). Samples for molecular and biochemical analyses were air dried and stored in the dark at ambient room temperature.

**Table 1:** List of the studied samples of *Apatococcus ammoniophilus*.

Voucher number	Coordinate	Date	Substrate		Country	Locality	BioSample accessions
US12909	56.016°N, 11.991°E	12-09-2018	Pinus mugo, bark	C	Denmark	Sealand, Melby Overdrev	
US12914	55.2716°N, 8.9539°E	31-03-2022	Salix sp.	B	Denmark	Jutland, Stensbæk Plantage	
US12915	55.1508°N, 8.3338°E	31-03-2022	Pinus sp.	C	Denmark	Rømø, Tvismark	
US12916	55.1508°N, 8.3338°E	31-03-2022	Pinus mugo	C	Denmark	Rømø, Tvismark	
US12917	55.9715°N, 11.7681°E	07-05-2022	Pinus mugo, bark	C	Denmark	Sealand, Rørvig, Korshage	
US12918	55.5141°N, 9.3980°E	28-07-2022	Prunus sp.	B	Denmark	Jutland. Kolding	
US12919	55.6202°N, 9.1051°E	28-07-2022	Picea sp.	C	Denmark	Jutland, Vorbasse	
US12920	55.6177°N, 9.0920°E	28-07-2022	Picea sp.,twigs	C	Denmark	Jutland, Vorbasse	
US12921	55.7151°N, 9.0476°E	28-07-2022	Prunus padus	B	Denmark	Jutland, Grene Sande	SAMN49111103
US12922	55.7294°N, 9.0398°E	28-07-2022	Picea sp., dead twigs	C	Denmark	Jutland, Grene Sande	
US12923	55.7294°N, 9.0398°E	28-07-2022	Pinus mugo, twigs and needles	C	Denmark	Jutland, Grene Sande	
US12924	55.7209°N, 9.0077°E	28-07-2022	Pinus nigra	C	Denmark	Jutland, Store Råbjerg	SAMN49111104
US12925	55.6702°N, 8.9605°E	28-07-2022	Pinus silvestris	C	Denmark	Jutland, Donslund, 10 km S of Grindsted	SAMN49111105
US12926	55.6702°N, 8.9605°E	28-07-2022	Picea sp., dead	C	Denmark	Jutland, Donslund, 10 km S of Grindsted	
US12927	55.6450°N, 8.9262°E	28-07-2022	Pinus sp., bark and twig	C	Denmark	Jutland, Baldersbæk Plantage	
US12928	55.6476°N, 8.9033°E	28-07-2022	Larix sp., dead twigs	C	Denmark	Jutland, Baldersbæk Plantage	
US12929	57.5927°N, 10.3734°E	13-09-2022	Pinus sp., dead twigs	C	Denmark	Jutland, Vendsyssel, Aalbæk Stene	
US12930	57.5828°N, 10.4117°E	12-09-2022	Pinus sp., dead twigs	C	Denmark	Jutland, Vendsyssel, Aalbæk Plantage	
US12933	57.5646°N, 10.3527°E	13-09-2022	Sorbus sp., bark	B	Denmark	Jutland, Vendsyssel, Råbjerg Mose	
US12934	57.4497°N, 10.3425°E	14-09-2022	Abies sp., twigs	C	Denmark	Jutland, Vendsyssel, Katsig Bakker	
US12935	57.4368°N, 10.4189°E	15-09-2022	Quercus sp., twigs	B	Denmark	Jutland, Vendsyssel, Åsted ådal	
US12936	54.9298°N, 9.5897°E	28-10-2022	Alnus sp., bark	B	Denmark	Jutland, South Jutland, Gråsten Skov	
US12937	55.1015°N, 9.4058°E	29-10-2022	Betula sp., bark	B	Denmark	Jutland, South Jutland, Lerskov	
US12938	55.0050°N, 9.3555°E	29-10-2022	Alnus sp., bark	B	Denmark	Jutland, South Jutland, Bolderslev Skov	
US12946	55.4453° N. 9.0386° E	22-02-2023	Picea abies, needles	C	Denmark	Jutland, 5 km SW of Vejen, Foldingbro	SAMN49111106
US12947	55.5456° N. 8.5793° E	23-02-2023	Scandosorbus intermedia, bark, N-side	B	Denmark	Jutland, 5 km SE of Varde.	SAMN49111107
US12948	55.5924° N. 8.4938° E	23-02-2023	Pinus mugo, dead twigs	C	Denmark	Jutland, 4 km S of Varde. Varde Øvelsesterrain.	SAMN49111108
US12949	55.5508° N. 8.3732° E	23-02-2023	Larix sp., dead twigs	C	Denmark	Jutland, 10 km NW of Esbjerg, Hjerting plantage	
US12950	55.5619° N. 8.3175° E	23-02-2023	Larix sp., bark	C	Denmark	Jutland, 14 km NW of Esbjerg, Marbæk plantage	
US12951	55.5568° N. 8.3079° E	23-02-2023	Pinus nigra, bark	C	Denmark	Jutland, 14 km NW of Esbjerg, Marbæk Strand	
US12952	55.6088° N. 8.2086° E	23-02-2023	Pinus sp., bark	C	Denmark	Jutland, 15 km W of Esbjerg, Mosevrå Kirke	
US12953	56.0526° N. 12.5500° E	20-04-2023	Salix sp., bark	B	Denmark	Sealand, Teglstrup Hegn, Skidendam	
US12954	56.0263° N, 9.2023° E	14-07-2023	Pinus sp., dead twig	C	Denmark	Jutland, Harrild Hede	
US12955	56.0263° N, 9.2023° E	14-07-2023	Calluna sp., twigs	B	Denmark	Jutland, Harrild Hede	
US12960	57.0343° N, 8.8641° E	08-08-2023	Picea abies, twigs and needles	C	Denmark	Jutland, Thy, Østerild Plantage.	
US12961	57.0090° N, 8.5105° E	09-08-2023	Picea sp., dead twigs	C	Denmark	Jutland, Thy, Vangså Klitplantage	
US12962	57.1452° N, 8.9811° E	11-08-2023	Pinus sp., dead twigs	C	Denmark	Jutland, Thy, Lild Strand	
US12966	55.5456° N. 8.5793° E	16-09-2023	Scandosorbus intermedia, bark, N-side.	B	Denmark	Jutland, 5 km SE of Varde.	
US12967	56.3156° N. 8.4810° E	15-09-2023	Salix sp., dead twigs	B	Denmark	Jutland, 13 km SW of Holstebro, Idom Hede	
US12968	56.9169° N, 8.4276° E	13-10-2023	Pinus sp., dead twigs	C	Denmark	Jutland, Thy, Stenbjerg Plantage, Præstens Bakker	SAMN49111109
US12969	56.9810° N, 8.4299° E	14-10-2023	Dead twigs	B	Denmark	Jutland, Thy, Thagårds Plantage	SAMN49111110
US12970	55.6109° N, 11.8833° E	26-10-2022	Fagus, twig	B	Denmark	Sealand, 15 km W of Roskilde, Bjergskov	
US13002a	55.7966° N, 9.1029° E	24-04-2024	Pinus sp.	C	Denmark	Jutland, 11 km NE of Grindsted	
US13002b	55.7966° N, 9.1029° E	24-04-2024	Betula sp.	B	Denmark	Jutland, 11 km NE of Grindsted	
US13003	55.8227° N, 8.8529° E	24-04-2024	Pinus sp.	C	Denmark	Jutland, 3 km SW of Sdr. Omme, Sønder Omme Plantage	
US13004	55.8227° N, 8.8529° E	24-04-2024	Pinus sp.	C	Denmark	Jutland, 4 km NE of Ølgod.	
US13005	55.5459° N, 8.5780° E	24-04-2024	Scandosorbus intermedia	B	Denmark	Jutland, 10 km SE of Varde, Rudholmvej	
US13006	55.7413° N, 9.3392° E	24-04-2024	Picea sp.	C	Denmark	Jutland, Bredsten Landevej	
US13007	55.4489° N, 8.3934° E	24-04-2024	Pinus sp.	C	Denmark	Fanø, Kikkebjerg Plantage	
US13011	55.7964° N, 9.1019° E	26-04-2024	Picea sp.	C	Denmark	Jutland, Langelund	
US13012	55.3108° N, 8.9229° E	26-04-2024	Pinus sp.	C	Denmark	Jutland, Årup Hede	SAMN49111111
US13013	55.3108° N, 8.9229° E	26-04-2024	Pinus sp.	C	Denmark	Jutland, Årup Hede	SAMN49111112, SAMN49111113 (subcultured agar sample)
US13014	55.3108° N, 8.9229° E	26-04-2024	Frangula sp.	C	Denmark	Jutland, Årup Hede	
US13016	55.9527° N, 11.7167° E	27-10-2019	Pinus sp., dead	C	Denmark	Sealand, Rørvig, Kabelhuset	
US13017	56.2921° N, 8.1372° E	19-09-2019	Pinus mugo, dead	C	Denmark	Jutland, Husby Klit.	
US13018	56.2682° N, 10.6735° E	29-01-2020	Pinus sp., dead twigs	C	Denmark	Jutland, Djursland, Stubbe Sø	
US13019	56.713° N, 8.430° E	23-03-2018	Larix sp., dead twigs	C	Denmark	Jutland, Thy, Dover Plantage	
US13021	55.5612° N, 11.8493° E	11-08-2024	Fagus sp., trunk	B	Denmark	Sealand, Lerbjerg Skov	
US13022	54.6565° N, 8.9529° E	20-09-2024	Picea sitchensis	C	Germany	Nordfriesland, Langenhorner Heide	
US13023	54.7565° N, 8.9889° E	20-09-2024	Picea sp.	C	Germany	Nordfriesland, Langenberger Forst	
US13024	54.2929° N, 8.8271° E	18-09-2024	Populus sp., trunk	B	Germany	Nordfriesland, Katinger Watt	
US13026	54.3197° N, 8.6124° E	17-09-2024	Betula sp.	B	Germany	Nordfriesland, St. Peter - Ording	
US13027	54.3197° N, 8.6106° E	17-09-2024	Pinus silvestris	C	Germany	Nordfriesland, St. Peter - Ording	
US13028	54.3197° N, 8.6106° E	17-09-2024	Pinus mugo	C	Germany	Nordfriesland, St. Peter - Ording	
US13029	54.5753° N, 9.0900° E	20-09-2024	Abies sp.	C	Germany	Nordfriesland, 3 km E of Bomsted.	
US13031	54.7565° N, 8.9889° E	20-09-2024	Picea sp.	C	Germany	Nordfriesland, Langenberger Forst	
US13032	54.3197° N, 8.6106° E	17-09-2024	Pinus silvestris	C	Germany	Nordfriesland, St. Peter - Ording	
US13033	55.5644° N, 11.8896° E	19-10-2024	Alnus sp., bark.	B	Denmark	Sealand. Hejede Overdrev, Avnsø	
US13041	57.6457° N, 10.4547° E	02-11-2024	Abies sp.	C	Denmark	Jutland, Vendsyssel, Bunken Klitplantage	
US13042	57.6457° N, 10.4547° E	02-11-2024	Pinus sp., dead twig	C	Denmark	Jutland, Vendsyssel, Bunken Klitplantage	
US13044	57.6348° N, 10.3860° E	02-11-2024	Pinus sp.	C	Denmark	Jutland, Vendsyssel, Råbjerg Hede, Lodskovvad Mile	
US13046	57.5724° N, 10.4211° E	04-11-2024	Pinus sp., twigs	C	Denmark	Jutland, Vendsyssel, Hvideklit	
US13051	55.3108° N, 8.9229° E	26-04-2024	Frangula sp.	B	Denmark	Jutland, Årup Hede	
US13052	54.9298° N, 9.5897° E	28-10-2022	Alnus sp., bark	B	Denmark	Jutland, Gråsten Skov	
US13053	54.3197° N, 8.6124° E	17-09-2024	Betula sp.	B	Germany	Nordfriesland, St. Peter - Ording	
US12871	58.1980°N, 11.4081°E	18-09-2021			Sweden	Bohuslän, Lysekils kommun, Skaftö sn, Islandsbergs huvud	
U. Søchting	52.3020° N, 5.7753° E	01-06-1989	Quercus twigs	B	Netherlands	Kootweik	
A. Pentecost	54.1098° N, 2.3590° E	06-08-2023	stone wall	A	UK	Yorkshire, Austwick	
A. Pentecost	54.5028° N, 3.1486° E	04-08-2023	Abies twigs	C	UK	Cumbria, Borrowdale	
A. Pentecost	54.5030° N, 3.1491° E	04-08-2023	N-facing fence pole	A	UK	Cumbria, Ashstead Fell	

(A), artificial substrate or stone; (B), broad leaved tree species; (C), coniferous tree species.

### Microscopy and attempts for culture

Microscopic observations were made using an Olympus BX60 microscope with Nomarski DIC optics; micrographs were taken with a Gryphax camera (Jenoptik) and processed with the corresponding Gryphax software (Jenoptik). Scanning Electron Microscopy was performed on freshly dried material from nature (US12952) that was attached to double-sided adhesive tape on a SEM stub and coated with gold–palladium. It was examined at 7 kV in a Jeol (Tokyo, Japan) JEM JSM 6335F field emission scanning electron microscope at the Zoological Museum, University of Copenhagen.

In an attempt to culture the filamentous alga, ten environmental samples were selected for culturing. Only one sample, US13013 from a *Pinus* twig, succeeded in growing, but the culture stopped growing after about 6 weeks. All further attempts to establish a stable culture failed. The US13013 material was kept in a dark and dry condition for about 1 week after collection. Material was scraped off the substrate with a sterile scalpel and carefully distributed on the surface of agarized (1.5%) Bold Basal culture medium with triple nitrate and vitamins (3NBBM + V, Available at: www.epsag.uni-goettingen.de; accessed 04 December 2024), a modification of the Bold 3 N medium of Starr and Zeikus (1993). The initial culturing was performed on agar plates maintained at 20°C under a light:dark (L: D) regime of 14:10 h and a photosynthetic photon flux density (PPFD) of 23 μmol m^−2^ s^−1^.

### DNA extraction, PCR, and sequencing

Algal material was scraped off the substrate (wooden twigs) into a sterile petri dish using sterilized scalpels and pooled into 100 μL reaction tubes containing the lysis buffer of the *Spin Plant Mini Kit* (Invisorb). The cells were carefully mechanically broken at 5,000 rpm twice for 30 s in the presence of equivalent amounts of 100–200 μm and 425–600 μm diameter glass beads (Sigma-Aldrich) using a PowerLyzer^®^ 24 instrument (MO BIO Laboratories, Inc., Carlsbad, California, USA). Genomic DNA was extracted with the same kit. DNA concentration was measured with an Invitrogen™ Qubit™ 3 fluorometer (Fisher Scientific). About 9–14 ng of the extracted DNA was used as a template for PCR amplifications in reaction mixtures of 50 μL volume. The 18S rRNA gene region was amplified with primer pair NS1 (White et al., 1990) and 18 L (Hamby et al., 1988) or NS1 and 1800R the reverse complement of primer 1800F (Friedl, 1996). For amplification of the ITS1–5.8S– ITS2 region with adjacent segments of the 18S and 26S rRNA gene regions served the primer pairs AL1500af (Helms et al., 2001) and LR3 (Vilgalys and Hester, 1990), as modified by Friedl and Rokitta (1997). PCR was performed with MyTaq™ DNA Polymerase (Meridian Bioscience) in a thermocycler Biometra TProfessional basic. The initial denaturation was at 95°C for 5 min, followed by 35 cycles of denaturation at 94°C for 45 s, annealing at 50°C for 45 s, extension at 72°C for 90 s, and a final extension at 72°C for 10 min. Amplicons were checked by agarose (1.5%) electrophoresis and purified with the MSB^®^ Spin PCRapace Kit (Invitek). The PCR products were sequenced at Microsynth Seqlab GmbH (Göttingen, Germany) using the PCR primers. The sequences were edited, and contigs assembled using the DNA sequence contig assembler software DNA Dragon (SequentiX - Digital DNA Processing, Klein Raden, Germany; Available at: www.dna-dragon.com accessed 04 December 2024). The 18S, ITS1, 5.8S, ITS2, and LSU regions were extracted from the long sequences with ITSx version 1.1b (Bengtsson-Palme et al., 2013; Rivers et al., 2018). The new sequences were compared with a cloned sequence, DZK5, which was obtained from the surface of a roof tile of a private house in a residential area within the city of Göttingen, Germany (51.558588 N, 9.939069E). The long sequences are available from the DDBJ/EMBL/GenBank databases under accession numbers PQ763401 - PQ763403.

### Paired-end ITS2 metabarcoding and sequence processing

For amplicon sequencing, the ITS2 rDNA region was targeted using the primer pairs ITS3-KYO2 (Toju et al., 2012) and ITS4 (White et al., 1990), which allow for the amplification of green algae and fungi. About 9–14 ng of the extracted DNA was used as a template for PCR amplifications in reaction mixtures of 50 μL volume. The PCR with barcoded primers [P7/P5 indices from Kircher et al. (2012)] and the PhusionTM High–Fidelity DNA Polymerase (Thermo Scientific) was done using the following program in a Biometra TProfessional basic thermal cycler: a first denaturation step at 98°C for 1 min followed by 25 cycles at 98°C for 45 s, annealing at 48°C for 45 s, and extension at 72°C for 30 s, and final extension at 72°C for 5 min. To capture and purify DNA fragments in the size range of 300–600 base pairs, MagSi-NGS^PREP^ Plus beads (Magtivio; Nuth, Netherlands) on an Ambion Invitrogen™ Magnetic Stand (Fisher Scientific), with a ratio of reagent/sample of 0.8, were used. Next, the samples were pooled, and the pools DNA concentration was adjusted to the requirements of the paired-end sequencing (≥ 50 ng/μL) using ethanol precipitation in the presence of ammonium acetate (Zeugin and Hartley, 1985). Illumina paired-end sequencing was performed at Novogene GmbH (Martinsried, Germany) on a Novaseq6000 platform on SP flow cell with v1.5 (500 cycles) chemistry (2 × 250 pair-end reads).

The high-throughput sequencing yielded 5,072,212 paired-end reads, which were demultiplexed using *cutadapt* [Version 4.9, (Martin, 2011)] with combinatorial dual indexing. We allowed a maximum error rate of 0.15 and a minimum overlap of 4 bases for index matching, resulting in 4,033,349 assigned reads. A more detailed description of the demultiplexing procedure can be found in the Supplementary file S1. Amplicon sequence variants (ASVs) were identified using *nf-core/ampliseq* [Version 2.11.0; (Straub et al., 2020)], a standardized bioinformatics pipeline implemented in *Nextflow* (Di Tommaso et al., 2017) within the *nf-core* (Ewels et al., 2020) framework. Primers were trimmed using *cutadapt*, and untrimmed sequences were discarded. Primer-free sequences were processed as one pool with DADA2 (Callahan et al., 2016) to remove *PhiX* contamination, truncate sequences (forward reads and reverse reads each at 220 bp, sequences shorter than this were discarded), discard sequences with more than two expected errors, merge read pairs and remove PCR chimeras. A total of 4,307 ASVs was obtained across all samples. Between 67.55 and 87.56% of reads per sample (average 82.4%) were retained. The ASV count table contained 3,241,892 counts, at least 75,953 and 746,194 per sample (average 294,717). Taxonomic assignment of ASVs was conducted using BLASTN [Version 2.14.0; (Altschul et al., 1990)] against the GenBank Nucleotide database (NCBI-GenBank Flat File Release 259.0 of December 15, 2023) using default parameters (Altschul et al., 1997). Taxonomic labels were assigned to ASVs using the consensus-based protocol of Rybalka et al. (2023). To ensure accuracy, we validated the taxonomic assignments through manual examination of consensus taxonomy, using normalized BLAST bitscore (*NB*) values as a quantitative criterion for acceptance (Rybalka et al., 2023). ASVs abundance data were filtered using R (Version 4.3.1, R Core Team, 2024) with *phyloseseq* (Version 1.46.0; (McMurdie and Holmes, 2014)) and *tidyverse* (Version 2.0.0; (Wickham et al., 2019)). After converting read counts to relative abundances, ASVs were retained if they exceeded a 0.1% relative abundance threshold in at least one sample (236 ASVs). A subset of 59 ASVs were clustered at a 98% sequence similarity threshold using VSEARCH (v2.29.1) (Rognes et al., 2016) after sequence subset extraction with *seqkit* [Version 2.9.0; (Shen et al., 2016)], with the most abundant ASV selected as representative for multi-ASV clusters and abundances aggregated within each cluster. A heatmap, computed with *tidyverse* and *phyloseq* in R (McMurdie and Holmes, 2013), to show the relative abundances of clustered ASVs. Colors represent relative abundance values transformed using the shrinkage function.

$f(x)=\frac{x+\varepsilon }{1+2\varepsilon }(\varepsilon =10^{-5})$ followed by a quantile logistic transformation to enhance the visibility of low-abundance values.

**Figure 3:**
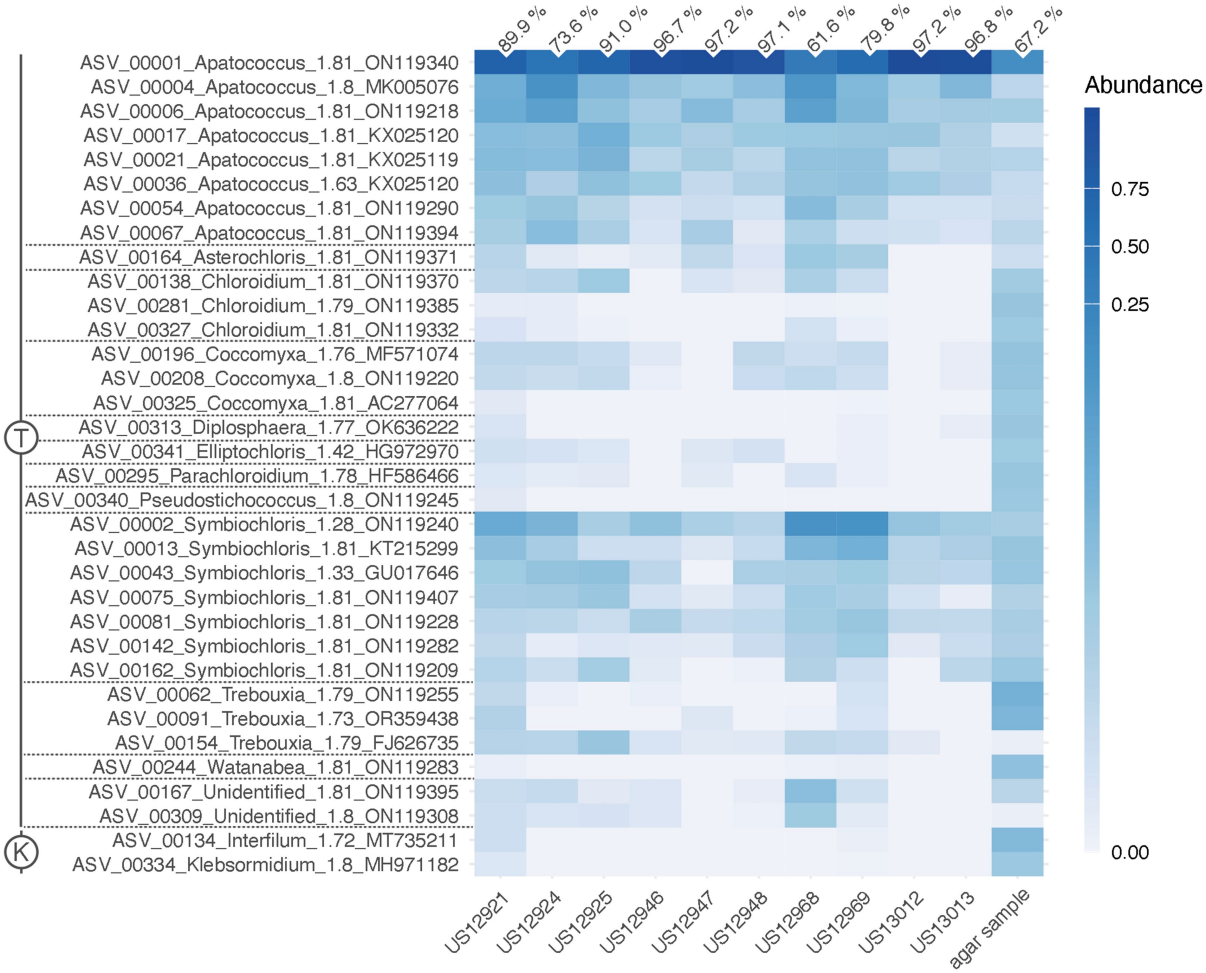
Heat map of relative abundances of the 34 clusters of green algal ASVs recovered by the ITS2 amplicon-based metabarcoding. It shows their distribution across the ten environmental samples and the subculture of sample US13013 on an agar plate (agar sample). The numbers on top are the relative abundances of ASV_00001 out of all algal reads per sample. An ASV ID is with its genus identification and the normalized score of pairing significance to its closest reference sequences (NB); (Rybalka et al., 2023). Scale, color brightness within the matrix indicates the relative abundance of sequence reads. T, Trebouxiophyceae (Chlorophyta); K, Klebsormidiophyceae (Streptophyta).

Of each of the 34 multi-ASV clusters, one representative, as listed in the caption of the heatmap (Figure 3), is available from the DDBJ/EMBL/GenBank databases under the accession numbers PQ784906 - PQ784939.

#### ITS2 phylogenetic analyses

The ITS2 region of all selected ASVs and reference sequences was annotated using the ITS2 database (Keller et al., 2009). ITS2 reference sequences were selected from (Zahradníková et al., 2017) and from the top BLAST hit for each ASV. Secondary structures of ITS2 sequences were predicted with RNAfold (Available at http://rna.tbi.univie.ac.at accessed Dec 4, 2024; Gruber et al., 2015) setting the temperature at 25°C and using minimum free energy (MFE) criteria. ITS2 sequences, including their secondary structures, were aligned with 4SALE 1.7.1 (Wolf et al., 2014). The maximum likelihood (ML) tree was estimated by 1,000 bootstrap replicates with the R script available at http://4sale.bioapps.biozentrum.uni-wuerzburg.de/mlseqstr.html in *phangorn* (Schliep, 2011) using one-letter encoded sequence-structure data exported from 4SALE (Wolf et al., 2014). Nine alternative topologies were constructed using the Interactive Tree of Life (iTOL) Version 6; Available at https://itol.embl.de accessed Dec 4, 2024; (Letunic and Bork, 2024) by repositioning the ASV_00001 lineage in different arrangements relative to the ML tree referred to as the “best tree.” A Shimodaira-Hasegawa (SH) test was conducted using the *SpeciesTopoTestR* (Adams and DeGiorgio, 2023) in R 4.4.1 (R Core Team, 2024), comparing each alternative topology against the best tree. Almost full 18S sequences were first aligned with a large set of reference sequences (143) that represented major lineages of the Trebouxiophyceae (Chlorophyta) using MAFFT (Katoh and Standley, 2013) and then curated, i.e., selecting regions in the multiple sequence alignment that are suited for phylogenetic inference, with BMGE (Criscuolo and Gribaldo, 2010) on the NGPhylogeny.fr web portal (Lemoine et al., 2019). The set of references included 26 different sequences longer than 1,000 base pairs representing the various lineages of the genus *Apatococcus.* The final dataset was 1747 nucleotides (columns) long with 753/545 variable/parsimony informative sites. A maximum likelihood phylogenetic tree was generated using IQ-TREE 2 (Minh et al., 2020). Branch support was assessed with the ultrafast bootstrap approximation [1,000 replicates, (Hoang et al., 2017)] implemented in IQ-TREE. IQ-TREE selected the TNe + R4 model to describe the best substitution pattern. The final tree was visualized and rooted using iTOL [Version 6; (Letunic and Bork, 2024)].

##### Metagenomic approach

For the metagenomic analyses, algal material from a *Larix* twig (US13019) was scraped off to obtain a powdery tissue from which total DNA was extracted using the Qiagen DNeasy Plant Mini Kit (Qiagen, Germantown). At the Research Resource Center at the University of Illinois, Chicago, the DNA was size-selected to target fragments with an insert size of 200 bp using a Pippin Prep Size Selection system (Sage Science, Beverly). A bead cleanup subsequently followed this process. The selected fragments were then used to create a sequencing library using the Nextera XT kit (Illumina, San Diego) before sequencing approximately 10 Gbp of 150 bp paired-end reads on an Illumina HiSeq4000.

To gather initial genomic information, we assembled all raw reads with SPAdes 3.11.1 (Prjibelski et al., 2020) using the default settings. All reads were inspected by using the program FastQC v0.11.3.^1^ Further, to improve the metagenomic assembly, we trimmed all reads with Trimmomatic v0.33 (Bolger et al., 2014), setting a quality threshold of 10 (LEADING:10 TRAILING:10) and a minimum read length of 25 bp (MINLEN:25). The surviving paired-end reads were used for another assembly with the metaSPAdes (Nurk et al., 2017) assembler implemented in SPAdes v3.13.0 and designed to perform better on metagenome assemblies. We used Quast v5.2.0 (Gurevich et al., 2013) to compare the quality of the two assemblies resulting from SPAdes and metaSPAdes. To receive coverage information for each scaffold in addition to the k-mer coverage provided by SPAdes, we used minimap2 v2.28-r1209 (Li, 2018) optimized for short reads (−ax sr -L --MD), and samtools v1.13 (Li et al., 2009) for sequence mapping, sorting, and counting of sequence coverage. All metagenomic raw reads were uploaded to NCBI with the accession number SRR32987805 under BioProject PRJNA1246228 and Biosample SAMN47770417.

We identified all contigs in the assembly that contain a 28S, 18S, 5.8S, and 5S ribosomal gene using the program barrnap v0.8 with the built-in “Eukaryota” database.^2^ In addition, we used the program SSU-ALIGN (Nawrocki, 2009), which identifies all contigs that contain the 18S ribosomal gene. We used all identified gene regions for a reciprocal megaBLAST (Altschul et al., 1990) search against NCBI’s non-redundant nucleotide (nt) database with Geneious Prime v2024.0. We then identified and retained all contigs classified as “Trebouxiophyceae” or “Klebsormidiophyceae” along with the top three results from each search for further review of each scaffold. To visualize the coverage and integrity of specific genomic regions, we mapped all metagenomic NGS reads back to the reference sequences using Geneious Prime v2025.0.3. Mapping was performed with Custom Sensitivity settings to ensure the highest possible stringency. The following parameters were applied: perfect matches only were allowed by setting the maximum mismatches per read to 0%, the minimum overlap identity to 100%, and disallowing gaps in the alignment. Ambiguous bases were limited to a maximum of 1. Reads were trimmed for paired-end overhangs, and the minimum mapping quality was set to 100 to include only high-confidence alignments. The mapping process did not include iterative reference extension or fine-tuning, focusing on fast and precise alignment.

##### Polyol analysis

For polyol extraction, 10–15 mg of the air-dried biofilm material was carefully scratched off various bark samples from *Pinus* species in Jutland, Denmark (US12923, 12,924, 12,925, 12,927). The resulting green powder was extracted in 70% aqueous ethanol (v/v) at 70°C for 4 h. After centrifugation at 8,300 × *g* for 5 min, 800 μL of the supernatant was evaporated to dryness and dissolved in 800 μL of autoclaved ultrapure water. After re-dissolution, vortexing and further centrifugation steps, the supernatant was stored in a 2 mL screw cap glass vial with silicone/PTFE septum (Wicom, Heppenheim, Germany) until high performance liquid chromatography (HPLC) analysis. HPLC was performed on an Agilent 1,260 Infinity HPLC system with RI-detector (Agilent Technologies, Santa Clara, CA, USA). Fast Carbohydrate Column (Bio-Rad, Feldkirchen, Germany) with a Carbo-Pb^2+^ guard column (Phenomenex, Aschaffenburg, Germany) was used for separation at 70°C and a flow rate of 1.0 mL min^−1^ at a pressure of 45 bar (Karsten et al., 1991). All polyols for calibration were purchased from Carl Roth (Karlsruhe, Germany). Polyol concentrations were calculated as μg per mg dry weight.

##### MAA analysis and structural elucidation

MS-grade acetonitrile was acquired from Merck (Darmstadt, Germany) and ammonium formate from Serva (SERVA Electrophoresis GmbH, Heidelberg, Germany). MS-grade formic acid was purchased from VWR International (Vienna, Austria). Ultra-pure water was prepared in-house with a Sartorius Arium 611 UV purification system (Sartorius AG, Göttingen, Germany). Standards of MAAs with confirmed chemical structure and purity were available from previous projects (Orfanoudaki et al., 2020; Zwerger and Ganzera, 2022). The dried algal biofilms were scratched off numerous bark and twig samples from different locations to reach sufficient biomass; they were pooled and milled using a rotating blade coffee grinder from Bosch (Munich, Germany) and sieved through a 710 μm steel mesh from Retsch (S/N 21241665, Body: 200 mm × 50 mm; Haan, Germany). An aliquot of the ground sample (m = 544.98 mg) was then extracted with HPLC-grade water (V = 10 mL) using ultrasonication (t = 15 min). Thereafter, the sample was centrifuged (RCF = 2,700 x *g*, t = 5 min) and the supernatant filtered through a paper filter with a particle retention of 10–20 μm from VWR International (Vienna, Austria). This extraction procedure was repeated four more times, and the filtered supernatants were combined. Using rotary evaporation under reduced pressure (T water bath = 40°C), the combined aqueous extract was dried. Any solvent residues were removed by freeze-drying to finally obtain 29.5 mg (5.4% dry weight) of dried extract. HPLC-DAD analyses were performed on an Agilent Technologies 1,260 Infinity II system equipped with a quaternary pump, vial sampler, column thermostat, and diode-array detector (Agilent Technologies, Santa Clara, USA). Separation conditions were according to the method described by Orfanoudaki et al. (2020), but the column oven was set to 12°C. The UHPLC analysis of the aqueous extract (c = 5 mg mL^−1^, solvent for dissolution = HPLC-grade water), a MAA standard mix and several MAA-containing fractions available from previous projects was performed on a Vanquish system (Thermo Scientific, Waltham, MA, USA) consisting of a quaternary pump, an auto-sampler, a column oven, and a variable wavelength detector connected to a Thermo Scientific Exploris 120 Orbitrap HRMS unit. Separation was carried out on a Phenomenex Luna Omega C18 100 Å column (100 mm × 2.1 mm; particle size = 1.6 μm) protected by a Phenomenex SecurityGuard ULTRA guard cartridge system (i.e., a UHPLC C18 pre-column). The mobile phase comprised water with 0.25% formic acid and 20 mM ammonium formate (A) and acetonitrile (B). The applied gradient was as follows: 0 min, 0% B; 10 min 0% B; 11 min, 90% B; 13 min, 90% B; 13.1 min, 0% B. Finally, the column was re-equilibrated with the original solvent composition (i.e., 0% B) for 19.9 min., which corresponds to a total run time of 33 min. The flow rate, column oven temperature, auto-sampler temperature, and injection volume were adjusted to 0.3 mL min^−1^, 17°C, 20°C, and 1 μL, respectively. The detection wavelengths were set to 330 and 350 nm, the data collection rate to 2.0 Hz, the response time to 2.0 s, and the peak with to 0.2 min. The system was controlled by Thermo Scientific Xcalibur 4.4 software. Calibration of the mass analyzer was done via the Thermo Scientific proprietary calibration mix and the respective automatic calibration function. The mass spectrometric parameters were as follows: heated-ESI ionization source, static spray voltage (positive and negative: 3500 V), sheath gas (N2): 30 arbitrary units, auxiliary gas (N2): 17 arbitrary units, sweep gas (N2): 0 arbitrary units. Temperature of the ion transfer tube and vaporizer was adjusted to 370 and 420°C, respectively. MS data (range 70–1,000 m/z) were recorded from 0 to 13 min with a resolution of 60,000 FWHM for MS1. The RF lens parameter was set to 70%. Data-dependent experiments were conducted with stepped collision energy mode and normalized collision energy type using HCD collision energies of 15, 30, and 45% at a resolution of 15,000 FWHM. The number of dependent scans was set to 3. The following selection of filters was employed: intensity threshold filter (1.0E5), dynamic exclusion (parent ions were placed in the exclusion list for 2 s after detection), isotope exclusion, charge state (perform dependent scans on singly charged precursors only), and apex filter (desired apex window: 75%). In addition, a specific exclusion list was created for the measurement using ultra-pure water as a background extract with an IODA Mass Spec notebook (Zuo et al., 2021).

## Results

### Distribution and environmental data

The filamentous alga was collected from living or dead bark of coniferous and deciduous trees and shrubs throughout Denmark (Figure 1), as well as from some locations in England, Sweden and the Netherlands (Table 1). Furthermore, it was collected from spruce and fir needles (Søchting et al., 1992), and from dwarf shrubs in highly ammonia-exposed habitats. The alga developed considerable filamentous biofilms (Figure 2), particularly in ammonia-producing farmland, which in Denmark is concentrated in the western part of the country (Figure 1). On twigs, it formed a continuous, light green biofilm on the upper side, while dominating the NNW-exposed sides of standing trunks. Generally, the alga formed a monoculture without being intermixed with lichens. Microscopic observation revealed a few cells of additional coccoid green algae, which were never dominant in the algal biofilms and comprised approximately one to 5 % of the algal community.

### Morphology

**Figure 4:**
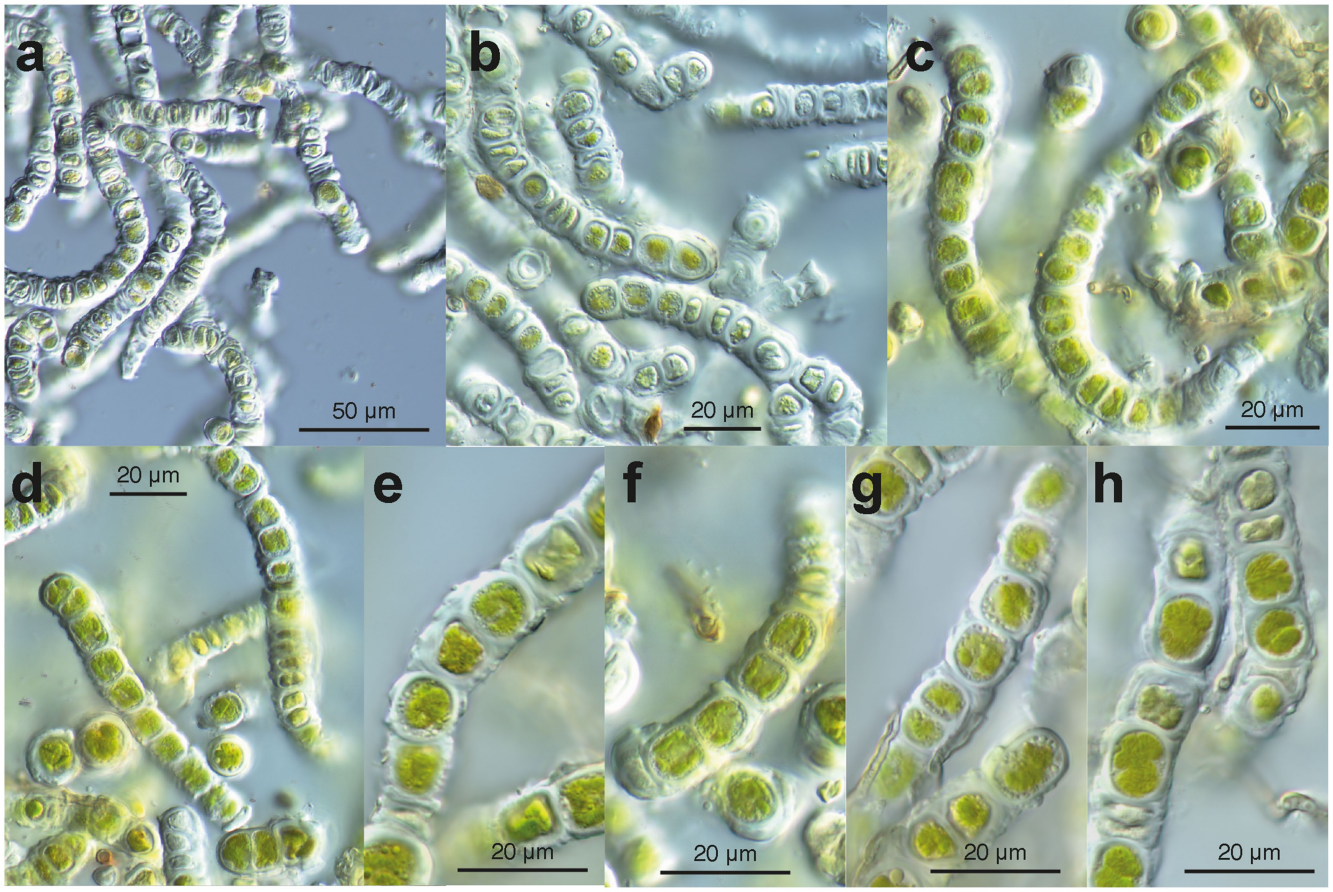
Microscopy of environmental samples of the filamentous *Apatococcus ammoniophilus*. **(a)** Low magnification overview of filaments, cells with thickened walls, and numerous empty cells. **(b)** Filaments consisting of two-celled units (autosporangia), thickened cell walls with remnants of ruptured old walls. Note the many pale non-vital cells. **(c)** Filaments of vital cells and uniseriate arrangement of two-celled units. Note the cap-like structure of the thickened wall (left filament). **(d)** Short fragments of filaments, cells with lobed chloroplast. **(e-h)** Closer view of cell structures and thickened cell walls. Note that storage droplets are close to the cell walls. **(e)** Units of two cells with remnants of old walls on the walls. **(f)** Two two-celled units in detail. Note cap-like wall structures between two units and lobed chloroplast. **(g)** Detail of a filament with two-celled units with thickenings of remnants of old cell walls. Filamentous fungus attached to the algal filament. **(h)** Vital cells (with lobed chloroplast) and damaged cells, filamentous fungus attached to the algal filament. **(a,b)**: sample US13027; **(c,d,f)**: sample US13029; **(e,g,h)**: sample US13028.

**Figure 5:**
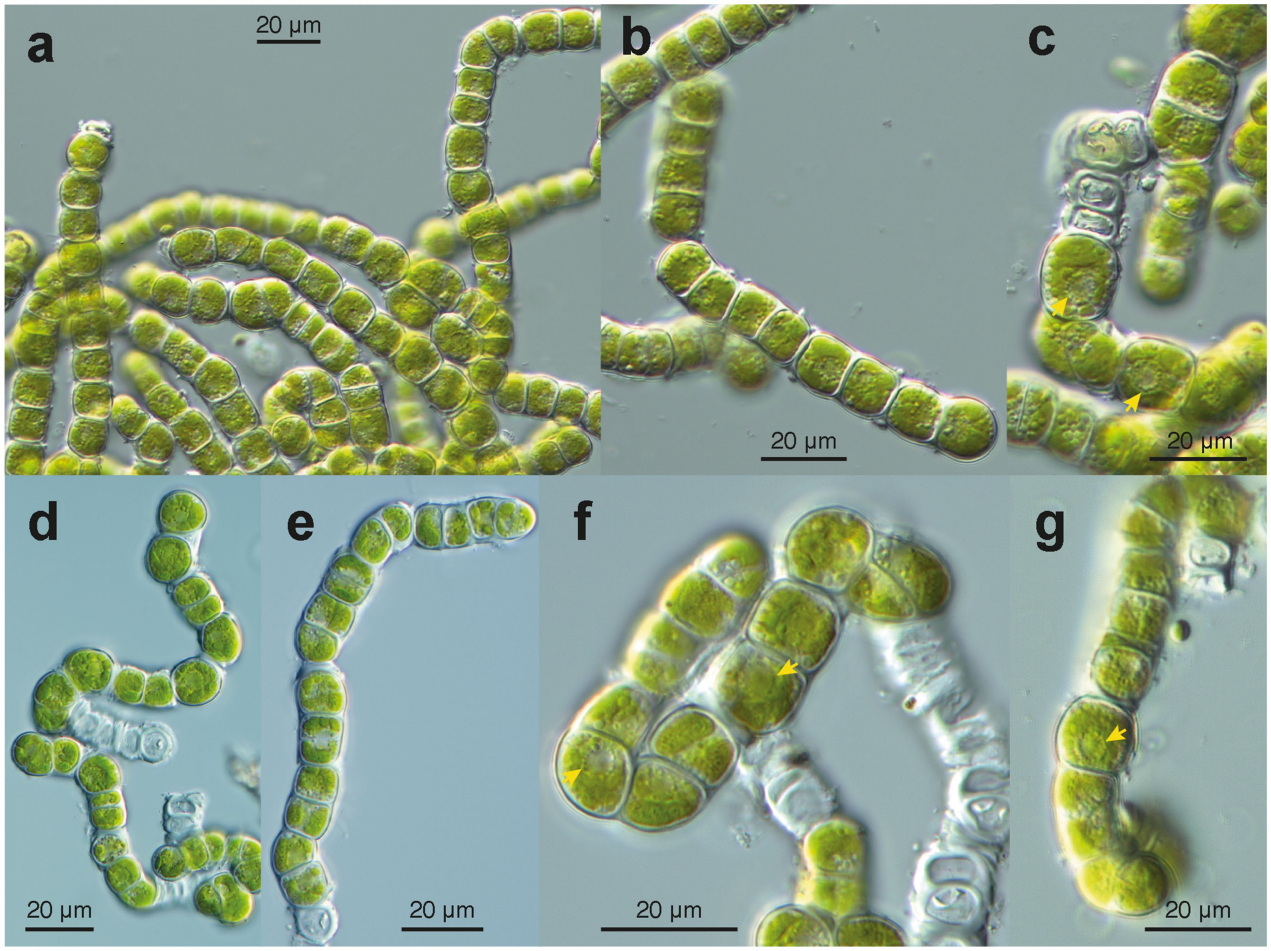
Microscopy of cultured filaments of *Apatococcus ammoniophilus* (sample US13013). **(a)** grown filaments without considerable wall thickenings. Note the filaments constitute a characteristic arrangement of pairs of two cells (autospores). Note cell package formation (lower right). Cells with a chloroplast of several flat lobes. **(b)**; Fragmented filament of a few two-celled units. **(c,f,g)**; Views at cellular details. Note collar of globules around nucleus with nucleolus (arrow heads). **(C)** Dividing cells in a filament and formation of cell packages. Flat chloroplast with several lobes. **(d,e)** Disintegrating filaments with cells that undergo division after about 3 weeks of maintenance on agar plates. Arrangement of pairs of cells (autospores). **(f,g)** Chloroplast with several flat lobes, collar arrangement of globules around the nucleus (arrowheads). **(f)** Note cell package formation (upper right). **(g)** Filament with cell package formation at its lower end.

In the uncultured environmental material, the filaments appeared to be formed by units containing two cells each (Figure 4). New walls encircled the two daughter cells formed within the same mother cell wall (Figures 4d–h). We interpret these observations as units that are expanding through cell growth; the old (mother) cell wall stretches and eventually ruptures, leaving cap-like remnants at both ends of a unit (e.g., Figures 4c,d,f,g). Small remnants of the ruptured old wall also persisted on the surface of the new wall (Figures 4e,g). The two-celled units remained attached at both ends, forming uniseriate filaments (Figures 4e–h). The filaments broke off between the units (Figures 4c,h), facilitating the reproduction of the alga. The cells contained a flat chloroplast with several lobes (Figures 4d–h). Droplets of storage products were found between the chloroplast and the cell wall (Figures 4e–g). Remarkably, many cells in the environmental material were not viable (Figures 4a,b); they exhibited damaged chloroplasts, protoplasts detached from the cell wall (Figure 4b), pale remnants of protoplasts within cells, and, ultimately, empty cell walls (Figures 4a,b). In parts of the filaments with empty cells, the walls appeared particularly thick, sometimes even swollen (Figure 4b); likely, they degraded very slowly. In culture, filaments consisting of units with two cells (autospores) each were observed (Figures 5a–g). Each two-celled unit may represent an autosporangium, and through their adhesion to each other at their ends, uniseriate filaments may be formed. The filaments easily disintegrated into shorter fragments comprising a few two-celled units (Figures 5d,e). Cap-like remnants of previous walls remained at the ends of the units and were kept distant by the growing filament. In the growing cultures, a tendency to form cell packages was also observed (Figures 5d,f,g). At some points in the filaments, the division plane shifted to the upright of the previous direction, thus forming initial cell packages (Figures 5c,f,g). Cultured cells were almost as long as wide (average 12.5 × 13.0 μm). The cultured cells exhibited a remarkable feature: a collar of globules, possibly representing tiny lipid droplets or large Golgi vesicles, around the nucleus (Figures 5c,f,g). The chloroplast was divided into several lobes, which were appressed to the wall (Figures 5f,g). No pyrenoids were seen. The combination of the latter two features is described as characteristic of the green algal genus *Apatococcus* (Gärtner and Ingolić, 1989). Unfortunately, after about 2 weeks of growth, the filaments ceased growing and disintegrated into short fragments of a few two-celled units (Figures 5d–g). The cells bleached, and empty walls became abundant (Figures 5d,f).

### Identification and composition of the algal community

The paired-end ITS2 metabarcoding analysis of ten environmental samples revealed that one ASV was predominant in a community of various green algae, accounting for 61.6 to 97.2% of all read counts per sample. The total number of algal reads per sample varied from 72,804 (sample US13012) to 590,258 (sample US12969). A total of 60 green algal ASVs, clustered into 34 ASV clusters, was found (Figure 3). Interestingly, 37 ASVs exhibited high similarities (NB ≥ 1.76) with 19 closest reference sequences assigned “uncultured alga” (accessions in the range ON119209 - ON119407). These were from a previous metabarcoding analysis of algal communities on tree bark (Dreyling et al., 2022). The consensus-based protocol that we applied could assign taxonomic labels to these ASVs. They represented 11 green algal genera from the class Trebouxiophyceae, two from the Klebsormidiophyceae, and two more remained unidentified (Figure 3). The predominant ASV_00001, along with seven other ASVs, represented *Apatococcus*. This is a significant finding because it suggests that the dominant mass-forming filamentous alga is a species of that genus. An agar sample grown from US13013 was also included, where the same ASV_00001 was predominant (67.2% of read counts). This proves that the same alga that dominated the filamentous algal masses in the environmental sample US13013 also developed on the agar plate. In addition, most other green algae ASVs appeared to be amplified through culturing on the agar plate (Figure 3), apart from the other *Apatococcus* ASVs, which may indicate that those algae are hard to culture. Though the two Klebsormidiophyceae ASVs, representing the filamentous genera *Interfilum* and *Klebsormidium*, were recovered by the amplicon-based metabarcoding approach from only a single environmental sample, US12921, they also developed on the agar sample, but at a lower relative abundance than ASV_00001. Thus, Klebsormidiophyceae had a rather low abundance in the studied samples.

### Phylogenetic position of the filamentous alga

**Figure 6:**
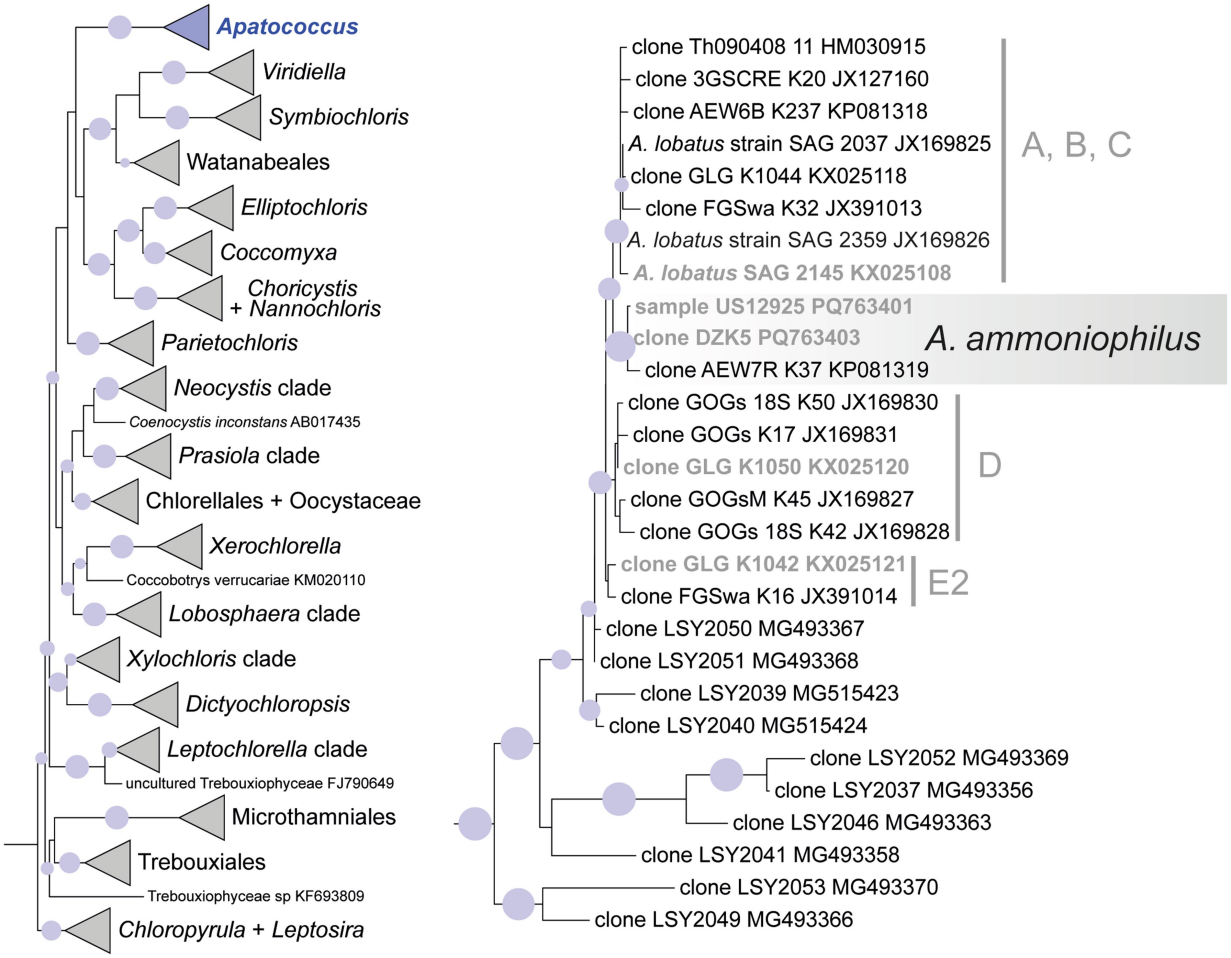
Maximum likelihood phylogenetic analysis (IQtree) of 18S rRNA gene sequences of *Apatococcus ammoniophilus* compared to 26 reference sequences representing *Apatococcus* and 117 other reference sequences representing major clades of the Trebouxiophyceae. The outgroup (two sequences of Nephroselmidales) has been pruned away from the graphic. Filled circles mark internal branches supported by >80% of 1,000 bootstrap replicates. The left figure shows the whole phylogeny with the *Apatococcus* clade highlighted. The right figure shows the internal structure of the *Apatococcus* clade with *A. ammoniophilus* highlighted. Marked lineages within *Apatococcus* are also resolved in the ITS2 rDNA phylogeny of Figure 7. Names in bold are those references containing 18S and ITS2 rDNA sequences.

Taking advantage of ASV_00001’s predominant presence in the ten samples selected for the metabarcoding approach, the nearly complete 18S rRNA genes from two of these samples were sequenced to enable the phylogenetic analysis of the filamentous alga. Long contigs of 2,976 and 3,020 base pairs were obtained from environmental samples US12924 and US12925 using the Sanger sequencing method. Both samples were also sequenced through the metabarcoding approach (Figure 3). The long sequences included the ITS1-5.8-ITS2 rRNA regions and the 5′-end of the 26S rRNA gene as well. The highly variable ITS2 of the two long contigs and that of ASV_00001 were identical; it was 211 base pairs long. It confirms that the 18S rRNA gene of the filamentous alga, which was dominant in the environmental samples, was sequenced. Direct comparison showed 100% sequence identity with the 18S rRNA gene and the ITS2 region of the cloned sequence DZK5, which we previously obtained from the surface of a roof tile in an urban region. Additionally, the ITS2 region of all three sequences matched the ITS2 region found in a sequence obtained from beech tree bark, accession number ON119340 (Dreyling et al., 2022). These findings indicate that the geographic distribution of the same genotype of the filamentous alga is broader than the samples we collected (Table 1) may suggest, and that it may colonize also other substrates besides tree bark. Phylogenetic analyses of the 18S rRNA gene placed the filamentous alga of the two environmental samples within a clade of the Trebouxiophyceae representing the genus *Apatococcus* (Figure 6). Thus, the analyses confirmed the sequence comparisons using the ASV_00001 sequence and proved the filamentous alga to be a species of *Apatococcus*.

**Figure 7:**
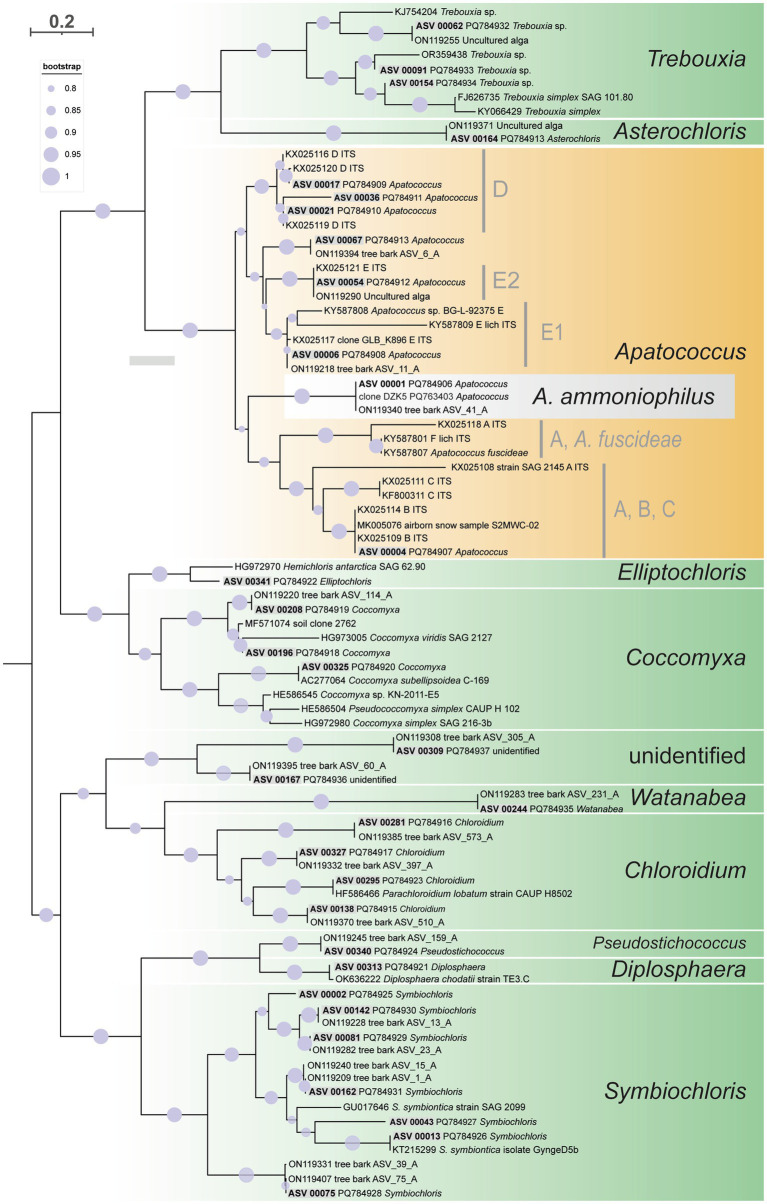
Maximum likelihood phylogenetic analysis based on ITS2 secondary structure models of the 34 multi-ASV clusters from the paired-end approach green algal ASVs (Trebouxiophyceae) recovered by the amplicon-based metabarcoding analysis, represented in Figure 3 and closest reference sequence. They are distributed on ten well-supported clades representing different genera of Trebouxiophyceae and one unidentified clade. Highlighted is ASV_00001 and its closest related sequences representing *Apatococcus ammoniophilus.* Filled circles mark internal branches supported by >80% of 1,000 bootstrap replicates.

The ITS2 of ASV_00001 and clone DZK5 were subjected to secondary structure-based phylogenetic analyses, including various reference sequences and the ITS2 sequences of the 34 ASV clusters as shown in Figure 3. The analyses confirmed the phylogenetic position of the filamentous alga in *Apatococcus*. Within the genus, the two new sequences and the sequence ON119340 formed an independent lineage (Figure 7) which suggests that the three sequences may represent a species of *Apatococcus* that has not been sequenced before. The *Apatococcus* clade was divided into subclades that roughly corresponded to the *Apatococcus* subclades discussed in Zahradníková et al. (2017). The subclade E of Zahradníková et al. (2017) was split into two lineages, E1 and E2, and the subclades A, B, and C (Zahradníková et al., 2017) were not separated from each other (Figure 7). The phylogenetic analyses also confirmed the identification of the other ASV clusters, i.e., their assignment to clades of Trebouxiophyceae genera was the same as obtained from the consensus-based protocol in the metabarcoding approach (Figures 3, 7). The two unidentified ASVs, ASV_00309 and ASV_00167, were together with their closest references, which have been assigned “Unidentified alga” (Dreyling et al., 2022) in a well-supported lineage, distinct from the other genera of Trebouxiophyceae and, therefore, remained unidentified also in the phylogenetic analyses. The lack of sufficiently close named reference sequence for those ASVs may indicate that the lineage may represent an yet unrecognized genus of Trebouxiophyceae. The phylogenetic position of the filamentous alga studied here within a clade that includes *Apatococcus* culture strains that exhibit a sarcinoid growth form, i.e., the culture strains SAG 2037, SAG 2145, and SAG 2359, was unexpected. Therefore, we tested the statistical significance of alternative tree topologies in which the filamentous alga, ASV_00001, occupied six other positions compared to the “best” tree from the maximum likelihood analysis presented in Figure 7 (Supplementary Figure S1). The two alternative tree topologies, where the ASV_00001 lineage was outside *Apatococcus* but adjacent to it (Supplementary figure S1), had high Delta log-likelihood (D-ML) values (*p* > 0.05), indicating they were significantly worse than the best tree and, consequently, unlikely to represent the true topology. The tests also rejected the other four alternative topologies with the ASV_00001 lineage positioned deeper within the Trebouxiophyceae, implying that any position of the alga other than inside *Apatococcus* was highly unlikely. Only the different arrangements within *Apatococcus* displayed low D-ML values (*p* < 0.05), suggesting they were plausible alternatives not significantly different from the best tree.

### Metagenomic identification

Independent of the amplicon-based metabarcoding approach, a metagenomic identification of the filamentous alga was achieved. Illumina sequencing of the algal material from environmental sample US13019 resulted in 36,938,013 paired-end sequences of a size of 150 base pairs, totaling over 11 billion base pairs. Although FastQC results confirmed good sequencing results, we used trimmomatic to improve the read data, which removed 5,286,889 paired-end reads after strict filtering, resulting in 31.651,124 paired-end reads for a metagenomic assembly. We first assembled all untrimmed reads using SPAdes but later attempted another assembly with metaSPAdes due to the metagenomic nature of our data. A comparison between SPAdes and metaSPAdes revealed that SPAdes generated more complete assemblies (Supplementary Table S1); therefore, we continued all further investigations with the contigs of the initial SPAdes assembly.

The program barrnap identified 196 contigs containing 26S, 18S, 5.8S, and/or 5S genes and the program SSU-ALIGN identified 158 contigs among the 482,599 contigs in the SPAdes assembly. When combining and deduplicating these results, we receive 324 contigs, indicating that both programs identify mostly different contigs with ribosomal genes. These contigs showed considerable variation in length and coverage, with an average length of 5,114.1 bp (SD = 16,773.47 bp), average k-mer coverage of 29.31x (SD = 94.06 x), and average per-site coverage of 446.54 x (SD = 2,917.59 x) (Supplementary Table S1). On the barrnap contigs, a total of 694 rDNA genes were identified and subjected to reciprocal BLASTn searches against NCBI’s non-redundant nucleotide (nt) database. This analysis identified 34 contigs of Trebouxiophyceae origin in the metagenomic assembly, with an average length of 14,967.35 bp (SD = 22,342.96 bp), average k-mer coverage of 98.25 x (SD = 205.28 x), and average per-site coverage of 283.39 x (SD = 459.89 x) (Supplementary Table S1). Barrnap did not identify any contigs with ribosomal genes of Klebsormidiophyceae origin. SSU-ALIGN identified 18 contigs with 18S ribosomal genes of Trebouxiophyceae origin, with an average length of 4,256.17 bp (SD = 5,781.62 bp), average k-mer coverage of 147.40 x (SD = 294.06 x), and average per-site coverage of 396.89 x (SD = 599.38 x) (Supplementary Table S1). SSU-ALIGN also identified one contig with parts of the 18S ribosomal gene of Klebsormidiophyceae origin of 306 bp length, a k-mer coverage of 2.11 x, and an average per-site coverage of 5.4 x (Supplementary Table S1). All identified contigs were ranked by k-mer and per-site coverage to assess genomic abundance and identify the algal species.

Seven contigs categorized as Trebouxiophyceae displayed high coverage, exceeding 100 x. Both SSU-ALIGN and barrnap identified 18S ribosomal genes on four of these contigs, which showed high alignment identity (97.9–100%) in a megaBLAST search with *Apatococcus* (NODE_230161, NODE_1182952, NODE_205589) or *Symbiochloris/Dictyochloropsis* (NODE_109413) (Supplementary Table S1). Additionally, barrnap detected the 5.8S ribosomal gene on NODE_205589 and NODE_109413. The 5.8S gene on NODE_205589 aligned with 98.1% identity to *Apatococcus*, while the gene on NODE_109413 matched 100% identity to *Symbiochloris/Dictyochloropsis*. Furthermore, barrnap identified the 28S ribosomal genes on three high-coverage contigs, NODE_233586, NODE_95039, and NODE_16933, which blasted with lower alignment identity (80.9 to 94.5%) to *Coccomyxa/Chlorella*, *Coccomyxa*, and *Lobospheara/Chlorella*, respectively (Supplementary Table S1). The highest coverage for all *Apatococcos*-identified contigs was recorded at 902.76 x, 751.16 x, and 677.58 x k-mer coverage, as well as for one of the *Coccomyxa* contigs containing 26S, which had 716.92 x k-mer coverage. All other identified contigs had k-mer coverages of 354.6 x or less. Using SSU-ALIGN, we identified one contig with parts of a *Klebsormidium* 18S ribosomal gene on contig NODE_1112260, which had a blast hit with a low identity of 90.8% and a low k-mer coverage of 2.11 x, while barrnap did not find any Klebsormidiophyceae ribosomal genes in the metagenome assembly.

The *Apatococcus*-identified contigs NODE_1182952, NODE_230161, and NODE_205589 were compared to the partial ribosomal repeat of samples US12924 (acc. no. PQ763402) and US12925 (acc. Number PQ763401) from the amplicon-based approach, which contained parts of the 18S, full ITS1, 5.8S, ITS2, and parts of the 26S rRNAgenes. The NGS sequences were consistent with those from the amplicon-based approach, including the highly variable ITS1 and ITS2 regions (Supplementary Figure S2). Mapping all identical metagenomic NGS reads to the sequence PQ763402 demonstrated continuous and uniform coverage across the reference sequence, with an average depth of 1,043.3 x (SD = 235.0 x), highlighting the robustness and depth of the *Apatococcus* ribosomal repeat region in the metagenome (Supplementary Figure S2).

### Polyols and mycosporine-like amino acids of the filamentous alga

**Figure 8:**
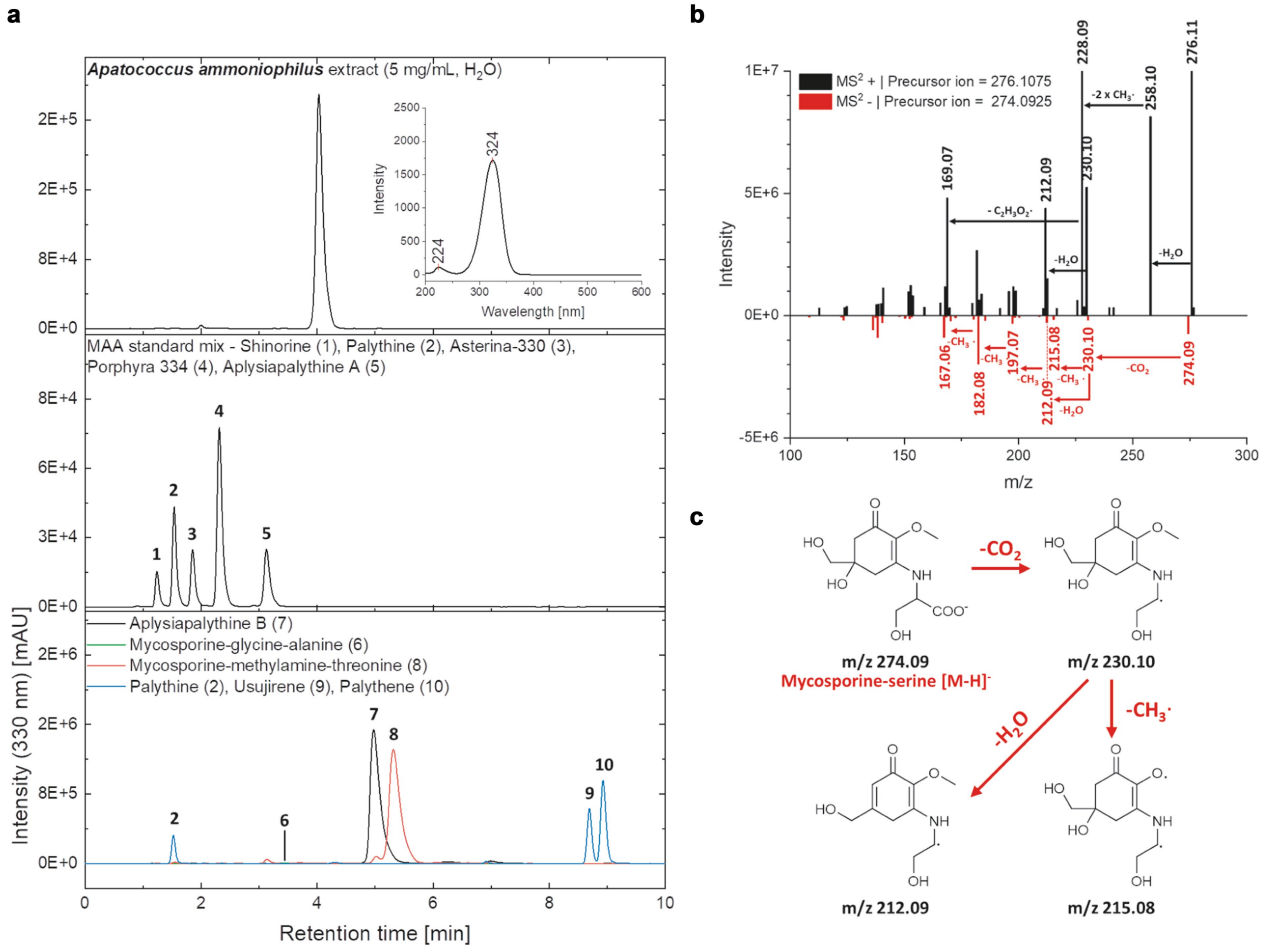
**(a)** HPLC-chromatograms of the *Apatococcus ammoniophilus* water extract (c = 5 mg mL^−1^), a standard mix with five mycosporine-like amino acids (MAAs) (shinorine (1 | RT = 1.24 min), palythine (2 | RT = 1.54 min), asterina-330 (3 | RT = 1.85 min), porphyra-334 (4 | RT = 2.31 min), aplysiapalythine A (5 | RT = 3.13 min)), and enriched fractions containing various MAAs aplysiapalythine B (6 | RT = 4.99 min), mycosporine-glycine–alanine (7 | RT = 3.50 min), mycosporine-methylamine-threonine (8 | RT = 5.31 min), palythene (9 | RT = 11.60 min), usujirene (10 | RT = 11.58 min) recorded at a wavelength of 330 nm. An on-line UV–Vis spectrum (*λ* = 200–600 nm) of the main peak in the *A. ammoniophilus* extract (RT = 4.03 min) is given in the chromatogram in the upper right section. **(b)** Mass spectra (MS2) of the main peak in the *A. ammoniophilus* extract (RT = 4.03 min) in positive (black) and negative (red) ESI mode. Characteristic fragments are highlighted with an arrow and the corresponding mass losses are indicated. **(c)** Hypothetical fragmentation pattern of mycosporine-serine, explaining several ions seen in the mass spectrum (B, negative ESI mode).

**Figure 9:**
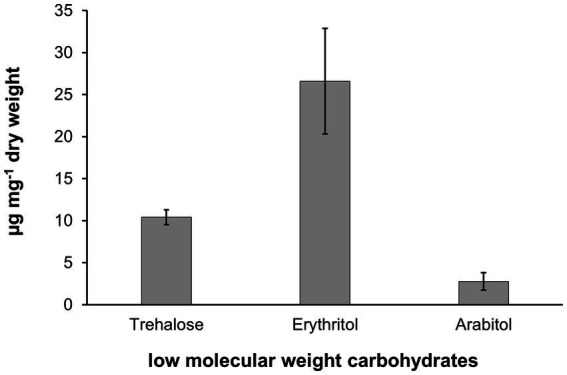
Low molecular weight carbohydrate pattern in *Apatococcus ammoniophilus* scratched off various bark samples from *Pinus* species in Jutland, Denmark (US12923, 12,924, 12,925, 12,927). Concentrations are given as μg mg^−1^ dry weight and represent mean values ± standard deviation (*n* = 3).

Biochemical analysis of low molecular weight carbohydrates of the biofilm samples dominated by the filamentous alga resulted in the identification of the polyols arabitol and erythritol, as well as the disaccharide trehalose. Erythritol is a C4 polyol and occurred as the quantitatively dominant carbohydrate with 26.6 μg mg^−1^ dry weight, while arabitol as C5 polyol was measured at 2.8 μg mg^−1^ dry weight (Figure 8). Trehalose concentration was determined at 10.4 μg mg^−1^ dry weight (Figure 9).

Chromatographic analysis of the biofilm water extract revealed the presence of a highly polar UV-absorbing compound that appeared as a prominent peak at a retention time of 4.03 min. and exhibited absorption characteristics consistent with those of mycosporine-like amino acids (MAAs) (Figure 8a). The online UV–Vis spectrum showed two maxima, at 224 and 324 nm, which fits well into their frequently stated absorption range of 310–360 nm (Karsten, 2008). Since the maximum at 324 nm is closer to that of MAAs with only one amino-acid substituent such as mycosporine-glycine (λmax = 310 nm) and palythine (λmax = 320 nm) and clearly below that of the aminocyclohexenimines porphyra 334 (λmax = 334 nm), usujirene, and palythene (both λmax = 359 nm), an aminocyclohexenone scaffold of the unknown compound seemed likely. Comparative liquid chromatographic studies showed that the retention time of the compound in the studied biofilm extract (RT = 4.03 min) differed significantly from the retention times of known MAAs listed in Figure 8a. The unknown compound eluted between mycosporine-glycine–alanine (7) and aplysiapalythine B (6) with retention times of 3.50 and 4.99 min. In the high-resolution MS experiments, the main peak of the biofilm extract was characterized by signals at *m/z* values of 274.0925 [M-H]- (ESI-) and 276.1075 [M + H] + (ESI+) (Figure 8b), suggesting a molecular formula of C_11_H_17_NO_7_ and consequently a neutral mass of 275 Da. The absorption properties, as well as the observed mass of the still unknown compound, suggest that it could be mycosporine-serine, which also complies with the hypothesis of an aminocyclohexenone core. Fragmentation reactions observed for the precursor ion in the positive mode (Figure 8B) include the elimination of water (Δ*m/z* = 18, 276.11→258.10), followed by the loss of two methyl groups (Δ*m/z* = 2 × 15, 258.10→228.09) and a C_2_H_3_O_2_∙moiety (Δ*m/z* = 59, 228.09→169.07). This fragmentation pattern is consistent with that of mycosporine-serine reported by Volkmann and Gorbushina (2006). Moreover, the fragmentation pattern of the negatively charged precursor ion (*m/z* 274.09 [M-H]-) can also be explained by reactions starting from mycosporine-serine, as illustrated in Figure 8c.

### Description of a new species

Both the amplicon-based and the metagenomic approaches identified *Apatococcus* as the most abundant sequence in the environmental samples, with the identified contigs from *Apatococcus* showing the highest coverage across the metagenomic assembly. This result demonstrates consistency across different methodologies and validates the dominance of *Apatococcus* in the sampled material. The exceptionally high coverage of these contigs further underscores the abundance of *Apatococcus* within the environmental sample. Additionally, the congruence of ribosomal gene regions, including the ITS1 and ITS2 sequences, supports the robust classification of *Apatococcus* as the predominant species in the microbial community analyzed. Therefore, we describe a new species of *Apatococcus* for the filamentous alga that prevails in the observed field material here:

### *Apatococcus ammoniophilus* Søchting, Friedl & Moestrup sp. nov.

#### Diagnosis

Thallus forming up to 2 mm thick, light-green mats consisting of filamentous, unbranched, ca. 10 μm thick, uniseriate aplanospores with flat, lobed chloroplast and lack of pyrenoid. Contains erythritol. Differs from all other known species of *Apatococcus* by formation of filamentous thallus. On living or dead bark of mostly coniferous trees (Figures 2–5).

#### Type

Denmark, West Jutland, 10 km SW of Varde, 55.5459°N, 8.5780°E, on *Scandosorbus intermedia* bark, collected on 24 April 2024, Søchting 13,005 (C); dried material deposited at Statens Naturvidenskabelige Museum, Copenhagen as no C-A-100671 (C-holotype; BM, GOET, LD, L-isotypes).

#### Description

Thallus forming up to 2 mm thick, light green mats consisting of filamentous, unbranched, uniseriate, 9–12 μm thick aplanospores formed by the adhesion of two-celled units. Cell lumina 6–7 μm thick, with 0.3 to 0.5 μm thick walls that are stratified and irregularly grooved. Cell lumina typically 3–9 μm long with a single flat chloroplast with irregularly lobed edges appressed to the cell wall; without pyrenoid. Cells with a large, central nucleus with a clearly visible nucleolus and a ring of small particles surrounding it. The cytoplasm close to the cell wall is granular and often filled with oil drops. The reserve product may be oil, not starch. Zoospores not observed, but filaments have intercalary growth and vegetative reproduction is achieved through disruption of filaments and formation of non-motile autospores. Cells containing erythritol.

In culture the filaments disintegrate shortly when the cell walls of previous generations dissociate, leaving behind units where the cells are grouped in pairs (two-celled units). A wall thickening may be present at one apical end of the two-celled units. Packages of cells (sarcinoid stages) can be present alongside the filaments and form through a cell division plane that is perpendicular to the one that results in the filaments; the filament cells are slightly elongated. Oil droplets vanish in culture. The cultured cells also feature a large central nucleus with a clearly visible nucleolus, surrounded by a ring (collar) of small particles.

#### Etymology

Named after its observed preference for strong atmospheric ammonia deposition.

#### Ecology

Terrestrial and subaerial, forming algal mats on the living and dead bark of coniferous and deciduous trees and shrubs, but may also grow on artificial hard substrates.

#### Distribution

Recorded so far from Denmark, Sweden, the Netherlands, Germany, and Great Britain. Very frequent in England, and also occurring in Scotland and Wales (A. Pentecost, in lit.). Most likely found in most of Northwestern Europe. The list of studied specimens is presented in Table 1.

#### DNA sequences

The ITS2 rDNA sequences are deposited in the NCBI GenBank database under accession number PQ784906 (ASV_00001), the 18S 3′-partial, ITS1, 5.8S, ITS2, and 26S 5′-partial sequences under the accession numbers PQ763401 (from voucher Søchting 12,925) and PQ763402 (from voucher Søchting 12,924). Metagenomic raw reads are also deposited at NCBI GenBank with the accession number SRR32987805 under BioProject PRJNA1246228 and Biosample SAMN47770417.

## Discussion

### Taxonomy

The filamentous alga studied here has earlier been assigned to *Hormidium crenulatum* Kütz., when a comprehensive morphological description with illustrations of cellular details was provided by Petersen (1915). Our observations of the studied filamentous alga, mostly from tree bark samples of Danish origin, fully match his descriptions. Therefore, our morphological observations on the fresh environmental (Figures 2-4) and the cultured material (Figure 5) leave no doubt that the alga recovered in our study here is the same as that of Petersen (1915). The first taxonomic description of *H. crenulatum* (Kützing, 1845) is incomplete because it refers only to the rough feature of the cell wall and the cell dimensions. In addition, Kützing’s description does not include drawings from which further cellular details could be inferred. *Hormidium crenulatum* Kütz. was lectotypified by Lokhorst in Lokhorst and Star (1985) because it was impossible to find any holotype material relating to *Hormidium crenulatum* Kütz. The lectotype in Leiden (L): “*L 939.67–834, annotated as “1/280” Hormidium crenulatum, Patavii, o*n wet and warm wall of a bath house, Padua, *leg. G. Meneghini” has not been available for microscopic examination.* Based on many cultured isolates, mostly from soil samples, Lokhorst (1996) published a new combination, *Klebsormidium crenulatum* (Kütz.) Lokhorst. Superficially, *K. crenulatum* resembles *A. ammoniophilus* as it can form filaments in culture with cell walls that appear “thickened and pronouncedly rough, (slightly) dented or crenulated” (Lokhorst, 1996). However, unlike the alga studied here and described by Petersen (1915), *K. crenulatum* regularly forms characteristic H-wall pieces. The *K. crenulatum* chloroplast is “plate- to girdle-shaped” and closely appressed to the cell wall; it possesses a pyrenoid with a prominent shell of starch grains (Lokhorst, 1996). In *K. crenulatum* the nucleus is within a central bridge of the cytoplasm, connecting opposite sides of the cell wall, a feature characteristic of many Klebsormidiophyceae (e.g., *Interfilum*, Mikhailyuk et al., 2008). Later, a culture strain, SAG 37.86, isolated from Alpine soil, was designated epitype of *K. crenulatum* to unambiguously link the name to a sequenced specimen (Mikhailyuk et al., 2015). The phylogenetic position of *K. crenulatum* as a distinct lineage within the genus *Klebsormidium* in the Klebsormidiophyceae (Streptophyta) was established (Mikhailyuk et al., 2015). Considering the morphological resemblance between *A. ammoniophilus* and *K. crenulatum*, the images of the filamentous *K. crenulatum* displayed in AlgaeBase (Guiry and Guiry, 2025) depict the same alga we studied here, but not *K. crenulatum* Kütz., underscoring the potential for confusion between the two. It follows that the alga we studied here, corresponding to the one reported by Petersen (1915), lacks a valid taxonomic name. Our study also uncovers that the alga is phylogenetically distant from *K. crenulata* because it is a member of the Trebouxiophyceae (Chlorophyta) and within the monophyletic clade representing the genus *Apatococcus*. Therefore, the alga needed to be described as a new species of *Apatococcus*. Gustavs et al. (2011) used the occurrence of specific polyols as an additional chemosystematic marker for different Trebouxiophyceae and showed that erythritol was exclusively detected in various *Apatococcus* strains. To the best of our knowledge, we are not aware of any other Trebouxiophyceae taxa containing this specific polyol, and hence the taxonomic position of *A. ammoniophilus* is well supported by this chemical marker. The only other algae known to also synthesize and accumulate erythritol are members of the aeroterrestrial genus *Trentepohlia*, which is in the Ulvophyceae (Chlorophyta) (Holzinger et al., 2023).

It may seem unexpected that a species of *Apatococcus* exhibits filamentous growth. Cell packages, i.e., a sarcinoid growth form, are typically associated with the species *A. lobatus.* The “parenchyma-like cell complex” or „multicellular mass “(Kinutani et al., 2015) or” aggregate building “(Gustavs et al., 2010; Gustavs et al., 2011) has often been described for *A. lobatus*. This species is a common member of algal communities on tree bark (e.g., Geitler, 1942; Vischer, 1960; Gärtner and Ingolić, 1989; Dreyling et al., 2022; Handa and Nakano, 1988). It is also very abundant in aeroterrestrial biofilms, particularly in urban areas, where it can cause extensive discolorations on walls and roofs (Rindi, 2007; Hallmann et al., 2013, 2016; Karsten et al., 2022). *A. ammoniophilus* filaments form through the adhesion of two-celled units (autosporangia). This feature corresponds to *A. lobatus,* where two autospores (immotile daughter cells) are formed within an autosporangium (Kinutani et al., 2015). The formation of only two cells by the division of the initially unicellular vegetative cells of *A. lobatus* has often been observed in earlier studies (Brand and Stockmayer, 1925; Geitler, 1942; Vischer, 1960; Gärtner and Ingolić, 1989). Characteristic of *A. lobatus* are cell packages that consist of three cells. The study of Kinutani et al. (2015) demonstrates that the *A. lobatus* three-celled packages arise from a delay in the formation of autospores, i.e., while two autospores are formed in one autosporangium, no division occurs in the other. In *A. ammoniophilus*, too, sarcinoid growth with packages of autosporangia can be observed in culture (Figure 5f), where the two daughter cells are formed at different times. However, in *A. ammoniophilus*, the cell division plane persists, resulting in chains of two-celled units that adhere to each other and result in intercalary elongation of the filaments. One could speculate that certain environmental conditions may suppress the change of the division plane, favoring filament formation.

One more remarkable feature connects *A. ammoniophilus* with *A. lobatus*: in the cultured cells, there is a centrally located ring of small particles (“Zentralkranz”) surrounding the nucleus (Brand and Stockmayer, 1925; Gärtner and Ingolić, 1989), which is clearly visible with Nomarski optics (Figure 5). According to the original diagnosis of the genus (Brand and Stockmayer, 1925, p. 351, figs 48, 50, 51), this feature distinguishes *Apatococcus* in culture from other terrestrial green algae found on tree bark.

### Adaptive traits of *A. ammoniophilus* for the aerophytic lifestyle

Aerophytic microalgae exhibit a variety of morphological, physiological, and biochemical traits for acclimation and adaptation to seasonally fluctuating environmental conditions (Holzinger and Karsten, 2013). Water availability and desiccation, along with high solar radiation, are key ecological factors determining the abundance and diversity of green algae in terrestrial biofilms. *A. ammoniophilus* contains three low molecular weight carbohydrates, i.e., arabitol, erythritol, and trehalose, with erythritol showing the highest concentrations (Figure 9). Both polyols are important protective compounds against desiccation-induced stress (Gustavs et al., 2010). However, erythritol and arabitol exhibit different chemical properties and energy requirements for biosynthesis, as erythritol is the smallest polyol with four carbon atoms, while arabitol consists of five carbon atoms. Trehalose is a disaccharide known to be formed particularly by desiccation-tolerant organisms, as it stabilizes biomolecules in a dehydrated state. The underlying mechanisms are still hypothesized rather than experimentally verified. These mechanisms include, for example, vitrification, as trehalose forms a glassy matrix that acts as a barrier, presumably physically shielding proteins or membranes from water loss (Kageyama and Waditee-Sirisattha, 2023).

Besides this interesting low molecular weight carbohydrate pattern, *A. ammoniophilus* also contained a probably new UV-absorbing MAA for Trebouxiophycean algae, as most members of this group contain the chemically elucidated MAA prasiolin (Hotter et al., 2018). Volkmann and Gorbushina (2006) published the only traceable report on mycosporine-serine, although the authors also refer to previous studies. As a consequence, the true nature of this interesting compound in *A. ammoniophilus* seems questionable, especially as its identification in the fruiting bodies of the fungus *Stereum hirsutum* was purely based on MS and UV–Vis data. Although the results presented herein (absorption maximum, retention time, mass, MS fragmentation pattern) strongly indicate the tentative identity of the unknown compound as mycosporine-serine, this still needs to be verified in additional experiments aimed at isolation and structural elucidation. This requires large amounts of clonal biomaterial, which makes a follow-up study necessary. In any case, final proof can only be provided by submitting unambiguous NMR and high-resolution MS data of the pure compound isolated from a clearly identified organism. The presence and properties of three low molecular weight carbohydrates, along with a rather unique MAA in *A. ammoniophilus,* well explain the biochemical basis for an aeroterrestrial lifestyle, i.e., the capability to synthesize anti-stress metabolites for compensation of desiccation and UV.

The filamentous morphology with intercalary growth may be advantageous as it allows a continuous and unlimited vertical increase of the filamentous mat. The formation of a thick mat of filaments will allow the species to expand over the substrate and overgrow more crustose competitors, resulting in almost monospecific ecological communities, but it may also result in shading of the lower part of the mat, eventually resulting in flaking off. Coccoid aerophytic algae have a low ability to take up free water (Barkmann, 1958), but are often successful on protected surfaces because they are able to absorb moisture from the air humidity. On the contrary, *A. ammoniophilus* grows on exposed surfaces on the twigs. It is able to instantly absorb moisture from dew or precipitation in the spongy mat, and additionally have a very large surface exposed to ammonia from the air. Interestingly, their light exposed habitat may also demand special protection by, e.g., MAA’s compared with their shade-preferring relatives. Field studies in combination with laboratory studies on clonal material are needed to investigate growth rates and other physiological traits under different environmental conditions, with a focus on ammonia requirements.

### *A. ammoniophilus* expansion in relation to increased ammonia deposition

**Figure 10:**
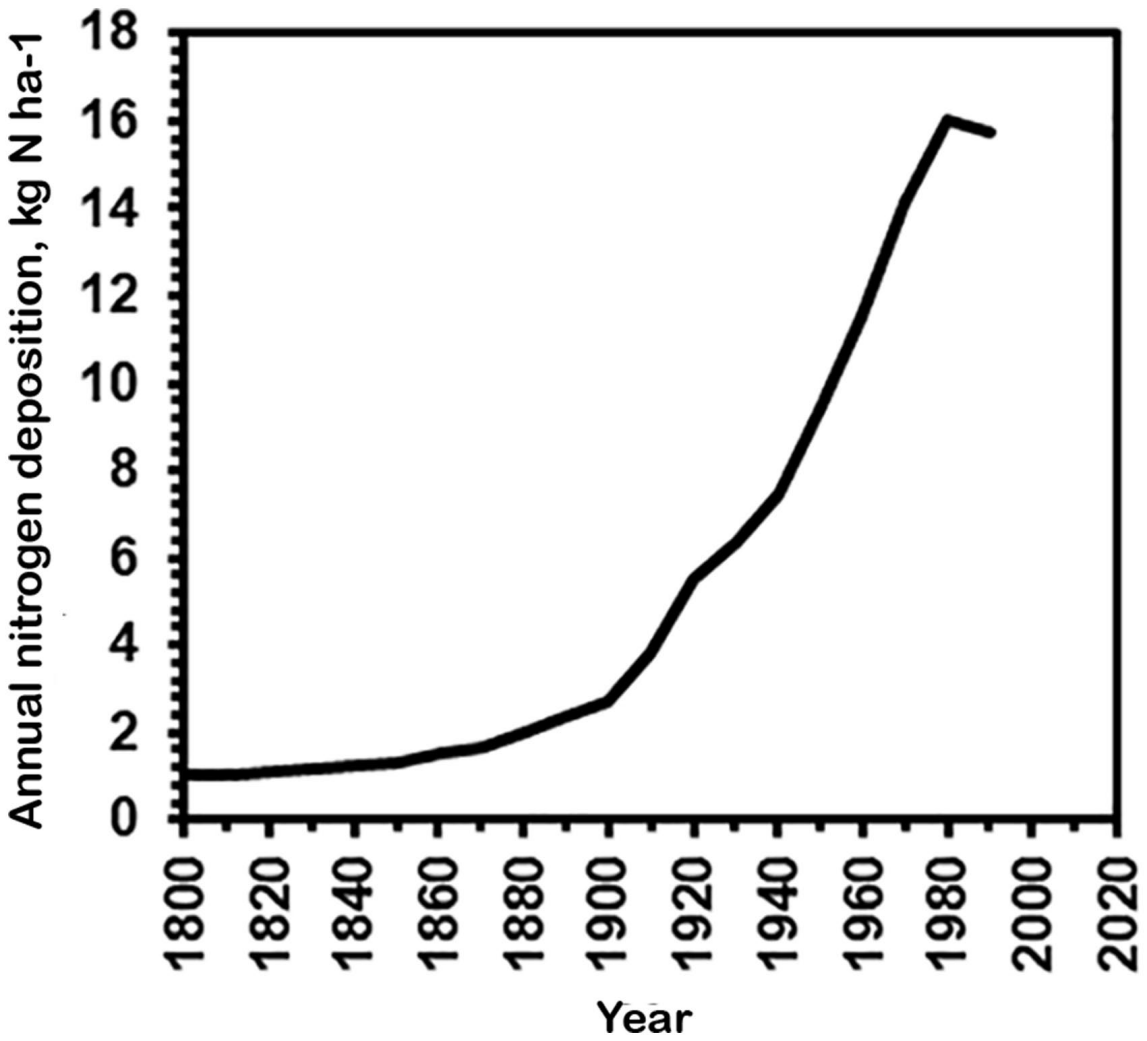
Average nitrogen deposition in Denmark 1800–1990 (kg N/ha) based on modelled estimates from Alveteg et al. (1998) and Ellermann et al. (2007).

*Apatococcus ammoniophilus* passed fairly unnoticed until it became abundant, most probably in response to decreasing acidification from acid rain in the seventies, and subsequent increasing ammonia deposition due to increased industrial farming. Estimates by Alveteg et al. (1998) and Ellermann et al. (2007) show a continuous average increase in nitrogen deposition from about 1 kg N ha^−1^ in 1800 to about 16 kg N ha^−1^ in 1990 (Figure 10). Over the subsequent 30 years nitrogen deposition has declined to about 12 kg N ha^−1^ in 2023, which is still 12-fold above pristine conditions (Ellermann et al., 2024). The species was unknown to the late Danish phycologist Dr. Tyge Christensen (Christensen, in litt), an extremely experienced phycologist and field biologist, indicating that it had expanded its occurrence only in recent years. Already Petersen (1915) noticed the preference of *A. ammoniophilus* for nitrogen enrichment. The locations of the studied samples of *A. ammoniophilus* were superimposed on the map of modelled total nitrogen deposition in Denmark in 2020 (Ellermann et al., 2021; EMEP, 2024) (Figure 1). However, the locations of the >100 *A. ammoniophilus* collections do not properly illustrate the substantial algal biomass abundance observed in the high-deposition regions in southern and western Jutland and the very sparse biomass in the low-deposition eastern part of the country, where *A. ammoniophilus* is only found if thoroughly searched for. The predominant occurrence in western Jutland is accentuated by sandy soil resulting in dominance of coniferous plantations, which appear to provide the most favorable substrate for *A. ammoniophilus.* Intensive farming areas in Denmark are those that have in recent years also received increased precipitation. However, there is no reason to expect this change to explain lichen disappearance; increased precipitation may even have increased wet deposition of ammonium.

Despite the lack of evidence from correlation data, careful field observations over many years and the abundance pattern concur that the occurrence and abundance of *A. ammoniophilus* might be related to atmospheric nitrogen deposition. Accordingly, we assume that it may even serve as a biological indicator of elevated ammonia deposition. The poor growth of *A. lobatus* in culture could be overcome by the addition of organic nutrient sources indicating mixotrophic behavior (Gustavs et al., 2016), which can also be expected for *A. ammoniophilus*.

### *A. ammoniophilus* occurrence and distribution

*Apatococcus ammoniophilus* is associated with other terrestrial green algae of the Trebouxiophyceae. The amplicon-based DNA metabarcoding revealed a distinct pattern with only slight variation across ten replicates, specifically nine tree bark samples and one coniferous needle sample (Figure 3). In all the samples, in addition to *A. ammoniophilus*, additional genotypes of *Apatococcus* were present. Those and most other genotypes recovered were the same as those revealed by a recent DNA metabarcoding-based study of tree bark microorganisms (Dreyling et al., 2024). It seems that there is a widespread terrestrial green algal community commonly found on tree barks in Central Europe, consisting of various genotypes and species from the Trebouxiophyceae, mainly from the genera *Apatococcus, Chloroidium, Coccomyxa, Symbiochloris,* and *Trebouxia*. *A. ammoniophilus* also belongs to that widespread community.

*Apatococcus ammoniophilus* has frequently been reported to one of us (US) by colleagues, who identified it as *K. crenulatum* based on field material observations from various locations in Great Britain. There, it is often viewed as a nuisance alga because of the significant biomass it forms on twigs of diverse trees lacking lichen cover. These observations support a broader distribution of the filamentous alga in Northern European regions with a more humid and cooler climate. The alga was also observed in England, where its appearance correlated with the disappearance of epiphytic lichens (A. Pentecost, *in litt*.). It is not studied whether the disappearance of lichens is caused by competition from *A. ammoniophilus* or is a direct effect of ammonia on the lichen thallus or its substrate, but when present *A. ammoniophilus* leaves very little space for lichens to colonize. From the United Kingdom, there are numerous observations on social media about the thick cover of a filamentous alga called *Klebsormidium crenulatum* on rocks and other solid surfaces in regions with heavy nitrogen deposition.^3^

Our DNA-based analyses, at the very fine level of sequence variants, revealed evidence of the same alga occurring in even more southern regions of Europe with a warmer and drier climate. The analyses recovered the same *A. ammoniophilus* genotype from locations presumably without elevated atmospheric nitrogen levels, i.e., a roof tile from a building in an urban environment and the bark of beech trees in forests. There, only inconspicuous green biofilms devoid of filamentous green algal growth were found.

## Conclusion

A microscopic green alga is forming significant biomass, becoming so obvious that it is regarded as a nuisance. This demonstrates that changes in terrestrial ecosystems, such as increasing deposition of eutrophicating nitrogen compounds from the atmosphere or climate warming, may strongly influence the algal communities exposed to open air. As an essential prerequisite for further studies on this phenomenon, our study provided insights into the phylogenetic position, taxonomy, and biochemical adaptive traits of a filamentous green alga that has expanded its occurrence only in recent years. We show that the alga, which was known under the name *Klebsormidium crenulatum* for more than 100 years, is, however, phylogenetically and structurally distinct from the latter species. We clarified the green alga’s phylogenetic position within the Trebouxiophyceae (Chlorophyta) and its close relationship within the genus *Apatococcus*. These efforts resulted in the description of a new species, *A. ammoniophilus*. There is consistent support for its classification across different DNA sequence-based methods and through an independent chemotaxonomic marker, i.e., the presence of the low molecular weight carbohydrate, erythritol. Future studies should focus on establishing culture strains of *A. ammoniophilus*, which, like its close relative *A. lobatus*, is difficult to maintain in culture. Furthermore, it is needed to experimentally test its apparent requirement for high levels of available ammonia.

## Data Availability

All sequence data generated from environmental samples as listed in Figure 3 and Table 1 using the paired-end approach (amplicon-based metabarcoding) have been deposited in NCBI in the Sequence Read Archive (SRA) under the BioProject accession number PRJNA1277513, with the BioSample accessions SAMN49111103 - SAMN49111113. One representative of each of the 34 multi-ASV clusters from the paired-end approach can be accessed from DDBJ/EMBL/GenBank databases with the accession numbers PQ784906 - PQ784939, the long 18S-ITS1, 2-26S rDNA sequences obtained from vouchers US12924, US12925, and clone DZK5 from the DDBJ/EMBL/GenBank databases with the accession numbers PQ763401 - PQ763403. All metagenomic raw reads can be accessed from DDBJ/EMBL/GenBank database with the accession number SRR32987805 under BioProject PRJNA1246228 and Biosample SAMN47770417.

## Footnotes

^1^ https://www.bioinformatics.babraham.ac.uk/projects/fastqc/

^2^ https://github.com/tseemann/barrnap

^3^ https://greenchristian.org.uk/filamentous-green-alga-on-walls-and-nitrogen-oxides-air-pollution/
