# Supplementary material for: Mass development of a filamentous and likely nitrophilous aerophytic green alga on tree bark: Apatococcus ammoniophilus sp. nov. (Chlorophyta, Trebouxiophyceae)
Source: Front Microbiol. 2025 Jul 23;16:1633308. doi: 10.3389/fmicb.2025.1633308 (PMC12325221; doi:10.3389/fmicb.2025.1633308)
Supplement: Supplementary file 1 [file Data_Sheet_1.docx]

**Supplementary figure S1**

**
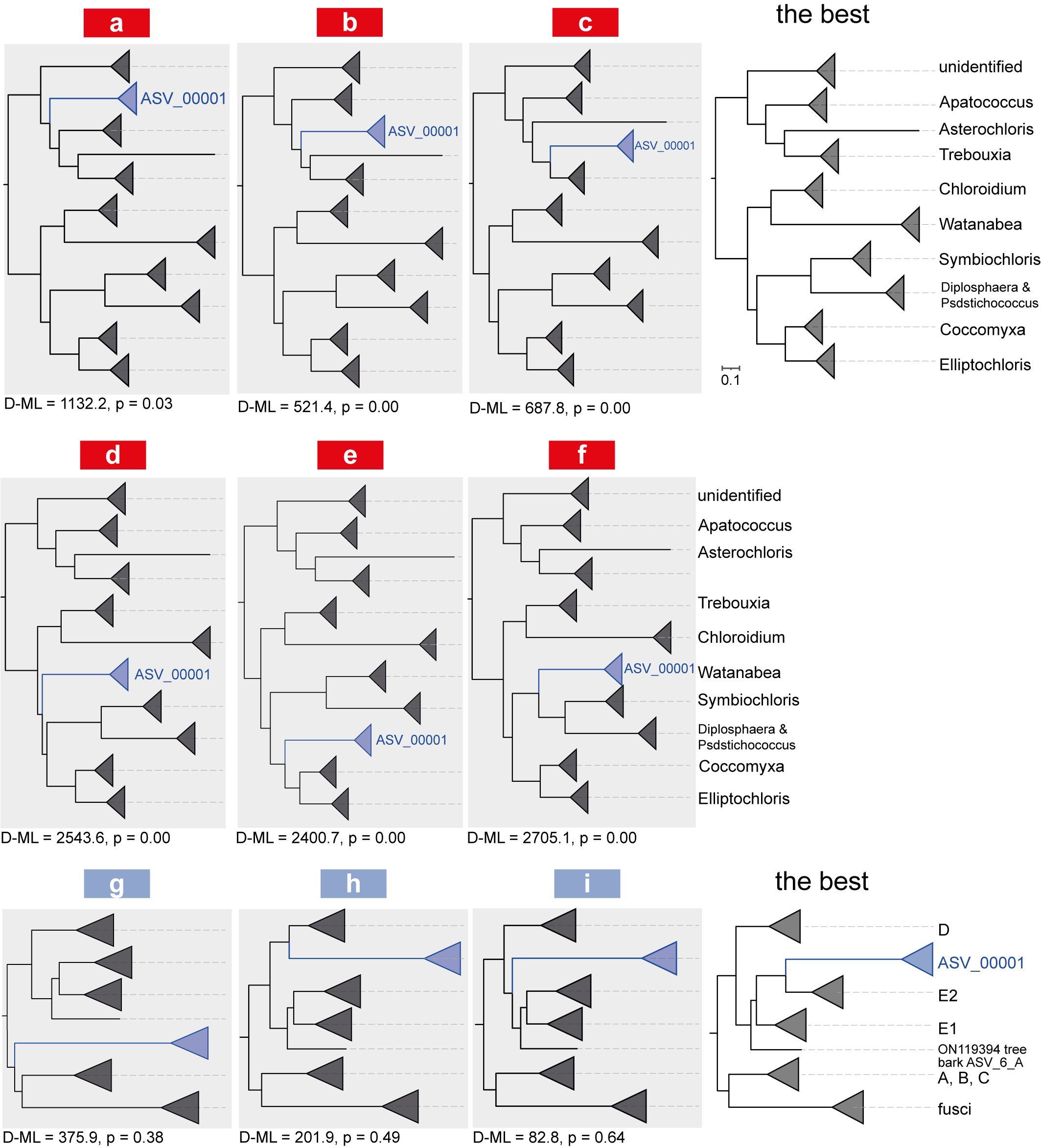
**

**Supplementary figure S1.** Testing of alternate topologies where the ASV_00001 lineage, representing *A. ammoniophilus*, is repositioned in different arrangements relative to the ML tree referred to as the “best tree” (see **Figure 7**) using the Shimodaira-Hasegawa (SH) test. The latter represents a phylogeny of all Trebouxiophyceae genera to which ASV_00001 was found associated in an algal community as revealed by amplicon-based metabarcoding (see **Figure 5**). **(a-f)** Six different positions of *A. ammoniophilus* among the various Trebouxiophyceae genera. Topologies a and b are for testing *A. ammoniophilus* being outside of *Apatococcus,* but a close relative of it. **(g-i)** Alternate topologies for testing *A. ammoniophilus* at different positions within *Apatococcus*. Alternate topologies highlighted in red have high Delta log-likelihood (D-ML) values (p>0.05), meaning they are significantly worse than the best tree and unlikely to represent the true topology. The topologies highlighted in blue have low Delta log-likelihood (D-ML) values (p<0.05), suggesting they are not significantly different from the best tree, making them plausible alternatives.
