# Supplementary material for: Mass development of a filamentous and likely nitrophilous aerophytic green alga on tree bark: Apatococcus ammoniophilus sp. nov. (Chlorophyta, Trebouxiophyceae)
Source: Front Microbiol. 2025 Jul 23;16:1633308. doi: 10.3389/fmicb.2025.1633308 (PMC12325221; doi:10.3389/fmicb.2025.1633308)
Supplement: Supplementary file 2 [file Data_Sheet_2.docx]

**Supplementary figure S2**


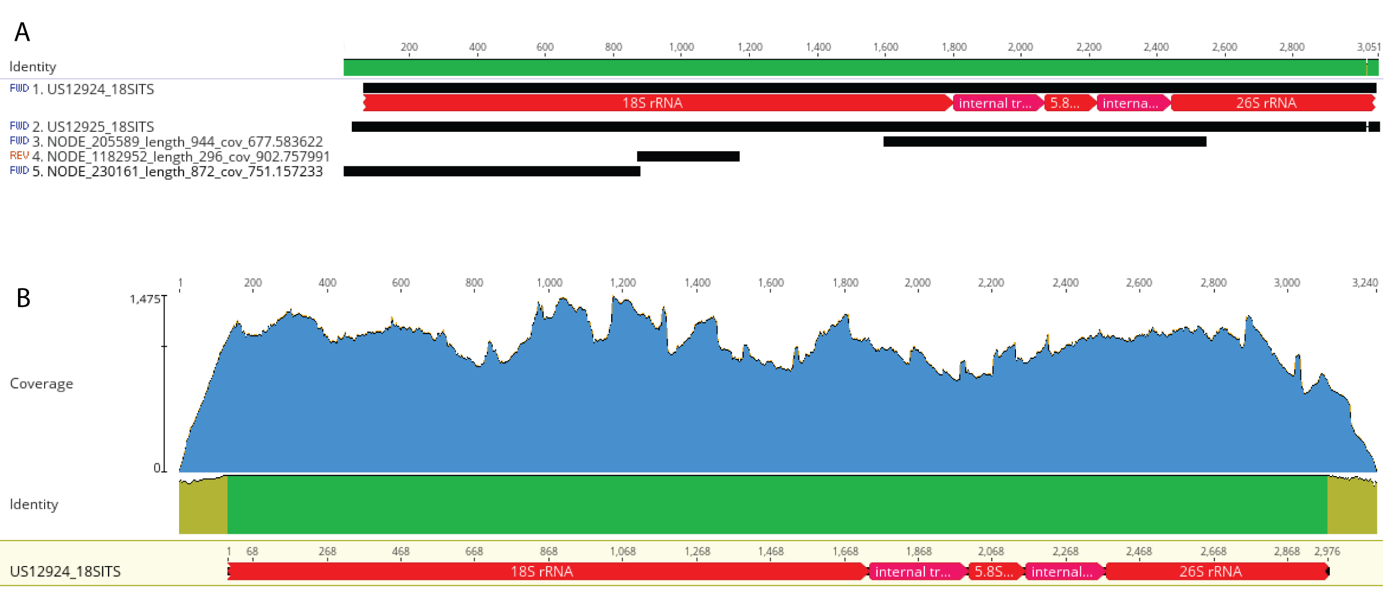


**Supplementary figure S2.** **(A)** The alignments display the rRNA regions from the NGS-assembled contigs (NODE_1182952, NODE_230161, and NODE_205589) mapped to the ribosomal repeat of clone US12924 (acc. no. PQ763402) and US12925 (acc. no. PQ763401) obtained from the amplicon approach. Reference sequences are shown at the top of each alignment, with annotations indicating rRNA regions (18S rRNA, ITS1, 5.8S rRNA, ITS2, and 26S rRNA). NGS contigs are shown below, with black bars representing the aligned portions. These contigs collectively reconstruct portions of the 18S rRNA, full ITS regions, and parts of the 26S rRNA. The sequence identity is visualized as a green histogram above each alignment, with green bars representing 100% identity. **(B)** Coverage plot showing the depth of NGS reads mapped to the ribosomal repeat of sequence US12924. The blue area represents the coverage, with peaks and troughs reflecting variations in sequencing depth across the reference sequence. This plot highlights continuous coverage across the ribosomal repeat, including the variable ITS1 and ITS2 regions, with an average coverage of 1,043.3x (SD = 235.0x), confirming robust sequencing depth for the assembled contigs. Sequence identity is visualized as a histogram above the annotation, where green bars indicate regions of 100% identity.
