# Supplementary material for: Mass development of a filamentous and likely nitrophilous aerophytic green alga on tree bark: Apatococcus ammoniophilus sp. nov. (Chlorophyta, Trebouxiophyceae)
Source: Front Microbiol. 2025 Jul 23;16:1633308. doi: 10.3389/fmicb.2025.1633308 (PMC12325221; doi:10.3389/fmicb.2025.1633308)
Supplement: Supplementary file 3 [file Data_Sheet_3.docx]

**Supplementary file S1**

Demultiplexing

Raw FASTQ files containing paired-end reads with attached barcodes and primer sequences were processed using our custom pipeline, available at https://github.com/daniel-nimptsch/ demultiplex-scripts. The pipeline utilizes *cutadapt* (Version 4.9; (Martin, 2011)) for combinatorial dual indices demultiplexing, *Python* (Version 3.21; (Van Rossum and Drake, 2009)) for scripting and *seqkit* (Version 2.8.2; (Shen et al., 2016) for quality control. The workflow begins with quality control of input files using *seqkit* stats, followed by demultiplexing, where unique index sequences on both forward (R1) and reverse (R2) reads are used to assign read pairs to their corresponding samples. The process retained primer sequences for subsequent steps. Demultiplexing parameters included a maximum error rate of 0.15 for index matching to allow for mismatch when considering our index length of 7. The *–no-indels* flag was used to prevent insertions and deletions in index sequences. Read pairs were discarded if either the forward or reverse index could not be identified (*–discard-untrimmed*). The pipeline generates required intermediate files, including barcode and primer pattern FASTA files, while tracking read counts throughout processing. Following demultiplexing, files were renamed from index-based to sample-specific identifiers, and an *ampliseq*-compatible sample sheet was generated for downstream analysis.

Martin, M. (2011). Cutadapt removes adapter sequences from high-throughput sequencing reads. EMBnet.journal 17. doi: 10.14806/ej.17.1.200

Shen, W., Le, S., Li, Y., and Hu, F. (2016). SeqKit: A cross-platform and ultrafast toolkit for FASTA/Q file manipulation. *PLOS ONE* 11**,** e0163962. doi: 10.1371/journal.pone.0163962

Van Rossum, G., and Drake, F.L. (2009). *Python 3 reference manual.* CreateSpace, Scotts Valley, CA.
